# η^2^-Alkene Complexes of [Rh(PONOP-^i^Pr)(L)]^+^ Cations (L = COD, NBD, Ethene). Intramolecular Alkene-Assisted Hydrogenation and Dihydrogen Complex [Rh(PONOP-^i^Pr)(η-H_2_)]^+^

**DOI:** 10.1021/acs.inorgchem.0c03687

**Published:** 2021-02-11

**Authors:** Alice Johnson, Cameron G. Royle, Claire N. Brodie, Antonio J. Martínez-Martínez, Simon B. Duckett, Andrew S. Weller

**Affiliations:** ∥ Chemical Research Laboratories, Department of Chemistry, University of Oxford, Oxford OX1 3TA, U.K.; ⊥ Department of Chemistry, University of York, York YO10 5DD, U.K.

## Abstract

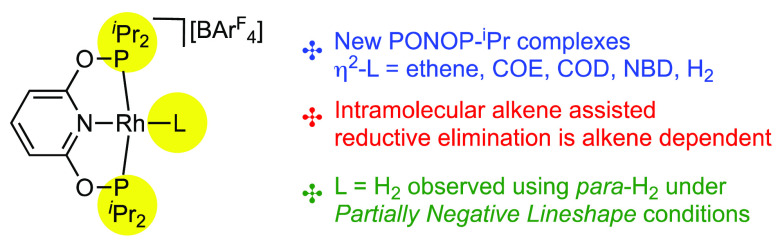

Rhodium-alkene complexes of the pincer ligand κ^3^-C_5_H_3_N-2,6-(OP^i^Pr_2_)_2_ (PONOP-^i^Pr) have been prepared and structurally characterized: [Rh(PONOP-^i^Pr)(η^2^-alkene)][BAr^F^_4_] [alkene = cyclooctadiene (COD), norbornadiene (NBD), ethene; Ar^F^ = 3,5-(CF_3_)_2_C_6_H_3_]. Only one of these, alkene = COD, undergoes a reaction with H_2_ (1 bar), to form [Rh(PONOP-^i^Pr)(η^2^-COE)][BAr^F^_4_] (COE = cyclooctene), while the others show no significant reactivity. This COE complex does not undergo further hydrogenation. This difference in reactivity between COD and the other alkenes is proposed to be due to *intramolecular* alkene-assisted reductive elimination in the COD complex, in which the η^2^-bound diene can engage in bonding with its additional alkene unit. H/D exchange experiments on the ethene complex show that reductive elimination from a reversibly formed alkyl hydride intermediate is likely rate-limiting and with a high barrier. The proposed final product of alkene hydrogenation would be the dihydrogen complex [Rh(PONOP-^i^Pr)(η^2^-H_2_)][BAr^F^_4_], which has been independently synthesized and undergoes exchange with free H_2_ on the NMR time scale, as well as with D_2_ to form free HD. When the H_2_ addition to [Rh(PONOP-^i^Pr)(η^2^-ethene)][BAr^F^_4_] is interrogated using *p*H_2_ at higher pressure (3 bar), this produces the dihydrogen complex as a transient product, for which enhancements in the ^1^H NMR signal for the bound H_2_ ligand, as well as that for free H_2_, are observed. This is a unique example of the partially negative line-shape effect, with the enhanced signals that are observed for the dihydrogen complex being explained by the exchange processes already noted.

## Abstract 2

η^2^-Alkene complexes [Rh(PONOP-^i^Pr)(η^2^-alkene)][BAr^F^_4_] [alkene = cyclooctadiene (COD), norbornadiene (NBD), ethene] are reported, for which only the COD complex undergoes significant onward reaction with H_2_ to form a cyclooctene (COE) complex, the reactivity is which is suggested to be due to intramolecular alkene-assisted reductive elimination. The putative product of H_2_ addition, [Rh(PONOP-^i^Pr)(η^2^-H_2_)][BAr^F^_4_], is made by an alternative route. Remarkably, enhanced signals for this dihydrogen complex are observed when *p*H_2_ is added to [Rh(PONOP-^i^Pr)(η^2^-ethene)][BAr^F^_4_], a unique example of the partially negative line-shape effect.

## 1 Introduction

**Scheme 1:**
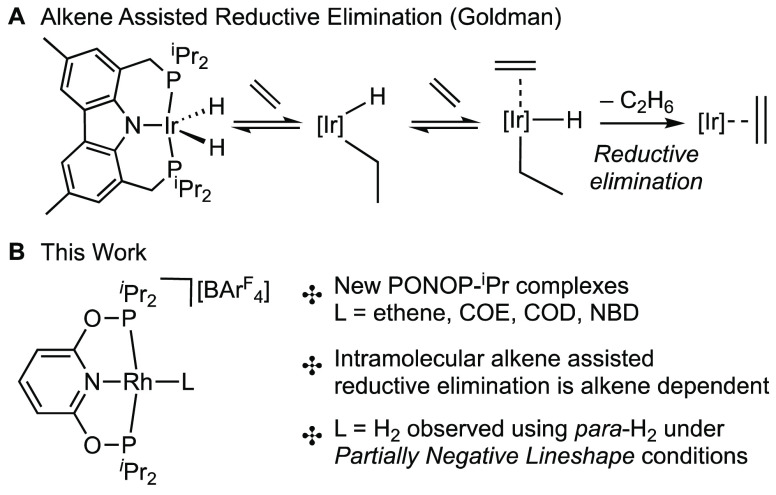
(A) Alkene-Assisted Reductive Elimination in Ir(carb–PNP)H_2_ Systems and (B) New PONOP-^i^Pr Systems and Intramolecular Alkene-Assisted Reductive Elimination in This Work

Pincer complexes of the group 9 metals (Co, Rh, and Ir) are used widely in catalysis.^1^ One important process that they are used in is the catalytic dehydrogenation of alkanes to form the corresponding alkene, often (although not exclusively^2^) using a sacrificial alkene to drive the reaction thermodynamics.^3,4^ An elementary step in this overall process when using a sacrificial alkene is the reductive elimination of an alkyl hydride at a metal(III) center. Reductive elimination of C–C and C–H bonds at d^6^ metal centers is often considered to operate through a five-coordinate intermediate, where a ligand dissociates prior to the reductive coupling event, if necessary.^5−8^ However, there are reports of reductive elimination being promoted by *association* of an external ligand, when steric and electronic factors allow.^9−13^ For example, Goldman has demonstrated that, for the neutral iridium(III), 16-electron, carbazolide-based pincer complex Ir(carb–PNP)H_2_,^14^ reaction with excess ethene ultimately gives ethane and Ir(carb–PNP)(η^2^-H_2_C=CH_2_). Here reductive elimination from an intermediate ethyl hydride is promoted by coordination of exogenous ethene (Scheme 1A), or even more strongly by H_2_ which then returns Ir(carb–PNP)H_2_ instead.

In this contribution, we report the synthesis of new cationic [Rh(PONOP-^i^Pr)(η^2^-alkene)][BAr^F^_4_] complexes [PONOP-^i^Pr = κ^3^-C_5_H_3_N-2,6-(OP^i^Pr_2_)_2_; alkene = cyclooctadiene (COD), norbornadiene (NBD), ethene; Ar^F^ = 3,5-(CF_3_)_2_C_6_H_3_]. When they are exposed to H_2_, a proposed *intramolecular* alkene-assisted reductive elimination leads to marked differences in both reactivity and selectivity in hydrogenation reactions, the outcome of which is dependent on the identity of the η^2^-bound alkene and, in particular, its ability to engage in bonding to the rhodium center with its additional alkene unit. We also report the independent synthesis of the dihydrogen complex [Rh(PONOP-^i^Pr)(η^2^-H_2_)][BAr^F^_4_] and some highly unusual observations during monitoring of the reaction of the corresponding ethene complex with *para*dihydrogen (*p*H_2_). This produces the dihydrogen complex as a transient product in which we, remarkably, observe enhanced ^1^H NMR signals for the bound H_2_ ligand, as well as for free H_2_, a consequence of the partially negative line-shape (PNL) effect that is in operation.^15^

## 2 Results and Discussion

### 2.1 Synthesis of [Rh(PONOP-^i^Pr)(η-L)][BAr^F^_4_]. η^2^ Binding of Alkenes

**Scheme 2:**
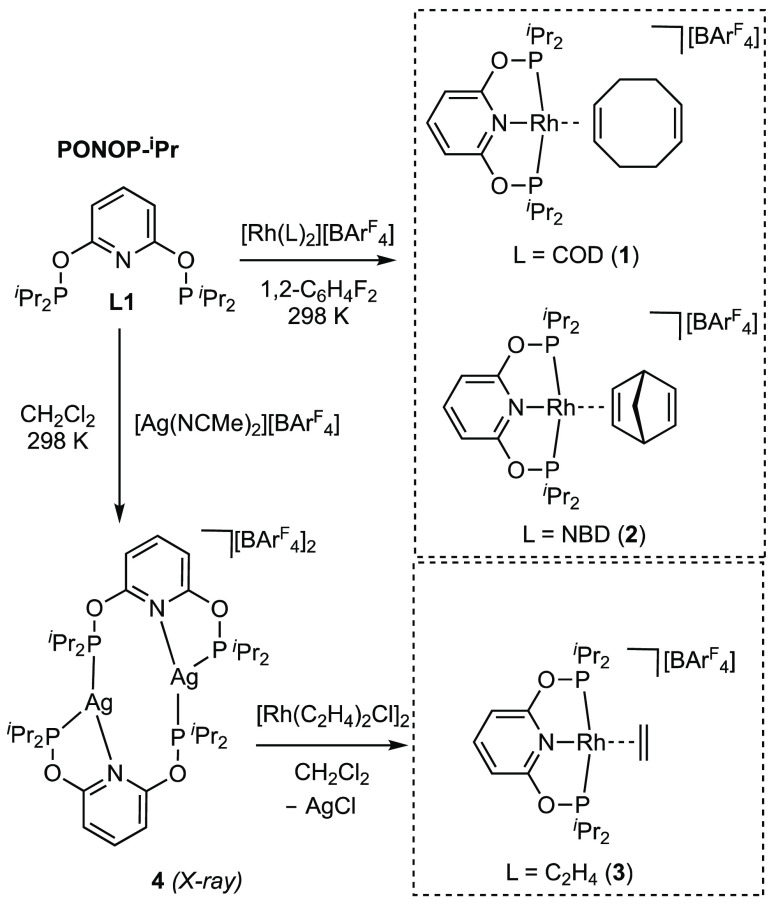
Synthesis of the New η^2^-Alkene [Rh(PONOP-^i^Pr)(alkene)][BAr^F^_4_] Complexes

We have recently reported on the mechanism of amine–borane dehydrocoupling using the cationic rhodium(I) catalyst [Rh(PONOP-^t^Bu)(η^2^-H_2_)][BAr^F^_4_] [PONOP-^t^Bu = κ^3^-NC_5_H_3_-2,6-(OP^t^Bu_2_)_2_].^16^ This dihydrogen complex has previously been reported to be made by H_2_ addition to the corresponding ethene adduct^17^ or to the equilibrium mixture of [Rh(PONOP-^t^Bu)_2_(μ-η^2^,η^2^-COD)][BAr^F^_4_]/COD and [Rh(PONOP-^t^Bu)(η^2^-COD)][BAr^F^_4_].^18^ Our initial aim was to explore how the now well-documented^19,20^ different steric demands of P^i^Pr_2_ versus P^t^Bu_2_ pincer arms could be harnessed in the synthesis and reactivity of pincer–dihydrogen complexes. We thus targeted synthesis of the precursor alkene complexes [Rh(PONOP-^i^Pr)(η-L)][BAr^F^_4_] (L = COD, NBD, ethene; Scheme 2). As we show, this was not an appropriate route to afford a dihydrogen complex. Alkene (or alkyne) adducts of group 9 pincer complexes are useful precursors in synthesis and catalysis, often activated by hydrogenation of the alkene^17,18,21−23^ ligand, and are also intermediates in alkane dehydrogenation reactions.^4,24^ More generally, while complexes of PNP-^i^Pr [PNP = κ^3^-NC_6_H_3_-2,6-(P^i^Pr_2_)_2_]^25−27^ and PONOP-^i^Pr^28−31^ complexes are well established, the only group 9 example of the latter is a cobalt complex.^32^

The ligand PONOP-^i^Pr (**L1**) was prepared in good yield (92%) and excellent purity (by ^1^H NMR spectroscopy) as a colorless oil by a slight modification of the published method.^29^ The addition to [Rh(COD)_2_][BAr^F^_4_] or [Rh(NBD)_2_][BAr^F^_4_] as 1,2-difluorobenzene solutions led to the isolation of new, analytically pure, complexes in good (67–75%) isolated yields, [Rh(PONOP-^i^Pr)(η^2^-COD)][BAr^F^_4_] (**1**) and [Rh(PONOP-^i^Pr)(η^2^-NBD)][BAr^F^_4_] (**2**), respectively. The ethene adduct [Rh(PONOP-^i^Pr)(η^2^-ethene)][BAr^F^_4_] (**3**) was prepared by a metathesis reaction using the previously unreported, structurally characterized, dimeric silver(I) adduct [μ-κ^3^-(PONOP-^i^Pr)Ag]_2_ (**4**; see the Supporting Information) and [Rh(η^2^-ethene)_2_Cl]_2_.

**Figure 1:**
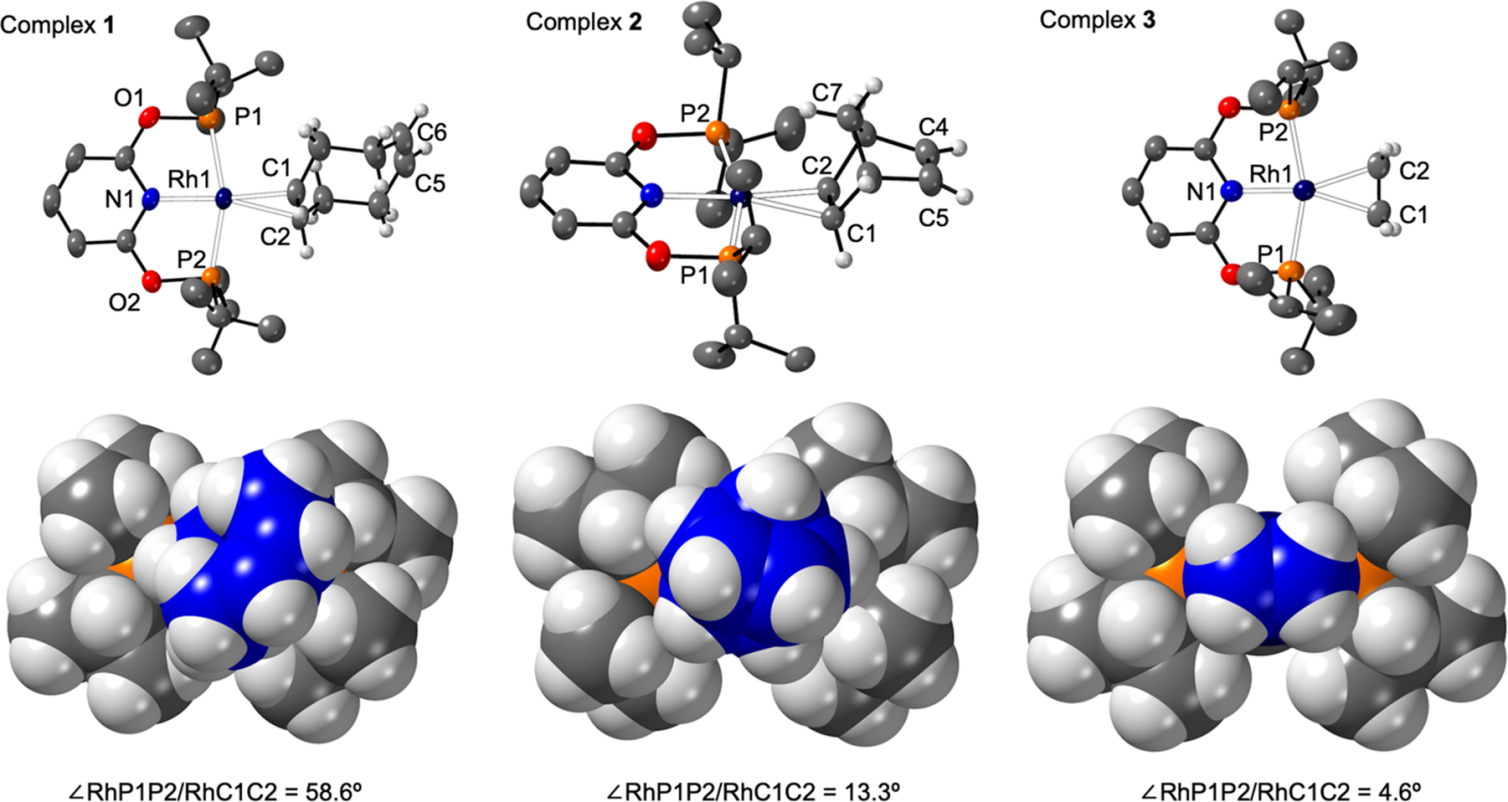
Solid-state structures of the cationic portions of complexes **1**–**3**. Displacement ellipsoids shown at the 50% probability level. Selected bond lengths (Å) and angles (deg) for **1**: Rh1–P1, 2.291(1); Rh1–P2, 2.249(1); Rh–N1, 2.050(3); Rh1–C1, 2.208(4); Rh1–C2, 2.222(4); C1–C2, 1.384(7); C5–C6, 1.309(9); P1–Rh1–P2, 160.20(4). Selected bond lengths (Å) and angles (deg) for **2**: Rh1–P1, 2.2522(8); Rh1–P2, 2.2712(8); Rh–N1, 2.053(3); Rh1–C1, 2.188(3); Rh1–C2, 2.175(3); C1–C2, 1.391(5); C4–C5, 1.329(6); P1–Rh1–P2, 160.03(3). Selected bond lengths (Å) and angles (deg) for **3**: Rh1–P1, 2.264(1); Rh1–P2, 2.2603(9); Rh–N1, 2.038(3); Rh1–C1, 2.165(5); Rh1–C2, 2.170(5); C1–C2, 1.326(8); P1–Rh1–P2, 160.27(4). Space-filling diagrams are shown at the van der Waals radii, with the alkene carbon atoms highlighted in blue.

These three new PONOP-^i^Pr complexes were characterized by single-crystal X-ray diffraction and solution NMR spectroscopy. Figure 1 shows the solid-state structures of the cations. Despite containing dienes, both **1** and **2** display η^2^-alkene bonding at the 16-electron pseudo-square-planar rhodium(I) centers. The C–C bond lengths in the coordinating, and noncoordinating, alkene groups are fully consistent with this description. Although the three structures are broadly similar, they differ in the orientation of the alkene ligand with respect to the RhP_2_N plane. The COD ligand in complex **1** lies toward being upright (*u*), while for NBD (**2**) and ethene (**3**), an in-plane (*ip*) conformation is seen. Both *ip* and *u* orientations of bound ethene have been observed for {ML_3_}-type group 9 PCP, POCOP, and PCNCN pincer systems, reflecting the interplay of steric and electronic factors at the metal center.^26,33−36^ Within the consistent set of complexes reported here, we suggest that steric effects dominate because the more locally compact ethene and bicyclic NBD ligands adopt a different orientation (*ip*) compared with the larger cyclic COD (*u*). The *u* orientation of the COD ligand is the same as that observed for [{Rh(PONOP-^t^Bu)}_2_(μ-η^2^,η^2^-COD)][BAr^F^_4_].^18^ It is interesting to note that COD and NBD ligands bind almost exclusively in a bidentate η^2^,η^2^-coordination mode to single metal centers,^37^ although rare examples of η^2^ binding are reported.^38−41^ The NBD ligand binds through its exo face.

Solution NMR data are broadly consistent with these solid-state structures. For complexes **1** and **2**, signals due to the unbound and η^2^-bound alkene units are observed (the latter resonances are upfield-shifted relative to the former) in the ^1^H NMR spectra, integrating to 2 H in each case (**1**, δ 5.64 and 4.83; **2**, δ 6.58 and 4.33). For the ethene complex **3**, a single environment is observed for the bound alkene (δ 3.09), integrating to 4 H. For all complexes, time-averaged *C*_2*v*_ symmetry for the {Rh(PONOP-^i^Pr)}^+^ fragment is indicated in the room temperature NMR spectra, as shown by signals indicating equivalent (but individually diastereotopic) ^i^Pr groups, a symmetric pyridine ligand, and a single ^31^P environment [with coupling indicative of a rhodium(I) center in such a pincer complex^17,18,27,42^]. Given the orientation of the COD (**1**) and NBD (**2**) ligands observed in the solid state, this suggests that a fluxional process operates in solution. A simple rotation^34^ of the bound alkene best explains the observed symmetry in solution rather than a dissociative process because there is no exchange between bound COD and free COD signals on the NMR time scale as determined by exchange spectroscopy (EXSY) NMR experiments; both bound and free alkene environments are observed, and diastereotopic NBD-bridged methylene signals are observed for **2** (δ 1.72 and 1.22). This lack of observable diene dissociation in solution is in contrast with the recently reported complex [Rh(PONOP-^t^Bu)(η^2^-COD)][BAr^F^_4_], which is in equilibrium with dimeric [{Rh(PONOP-^t^Bu)}_2_(μ-η^2^,η^2^-COD)][BAr^F^_4_] in solution via dissociation of COD, which preferentially crystallizes as the COD-bridged dimer.^18^ These differences are likely due to the different steric requirements of PONOP-^t^Bu versus PONOP-^i^Pr.^19,20^ Such differences carry over into the reactivity with H_2_ as discussed next.

### 2.2 Stoichiometric Hydrogenation in Solution. Role of the Distal Alkene as an Intramolecular Assisting Ligand

**Figure 2:**
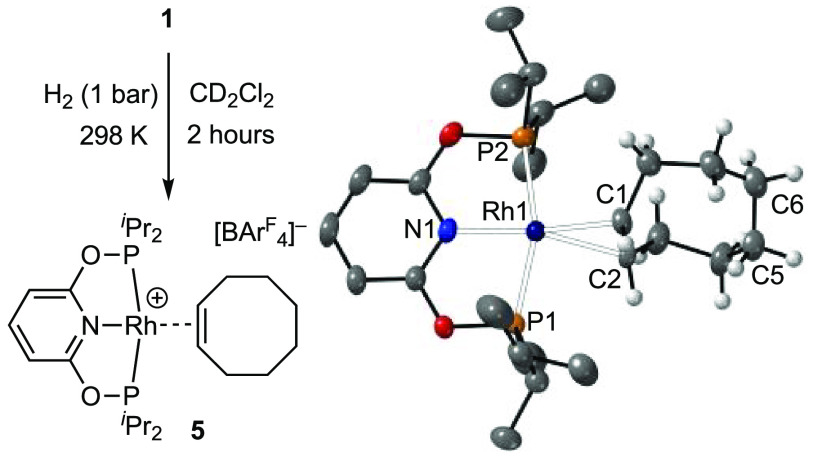
Synthesis and solid-state structure of the cationic portion of complex **5**. Displacement ellipsoids shown at the 50% probability level. Selected bond lengths (Å) and angles (deg) for **1**: Rh1–P1, 2.2537(6); Rh1–P2, 2.3028(6); Rh–N1, 2.060(2); Rh1–C1, 2.206(2); Rh1–C2, 2.210(2); C1–C2, 1.360(4); C5–C6, 1.529(5); P1–Rh1–P2, 160.38(2); ∠RhP1P2/RhC1C2, 69.8°.

The addition of H_2_ (1 bar) to a CD_2_Cl_2_ solution of complex **1** resulted in the relatively slow (2 h) but clean conversion to a complex in which partial, but selective, hydrogenation of COD to cyclooctene (COE) had occurred, to give [Rh(PONOP-^i^Pr)(η^2^-COE)][BAr^F^_4_] (**5**; Figure 2).^43^ Selective hydrogenation of a single C=C unit is shown by a single, relative integral 2 H, alkene resonance being observed in the ^1^H NMR spectrum (δ 4.65). The solid-state structure, as determined by single-crystal X-ray diffraction, shows a well-ordered COE ligand with bond lengths fully consistent with this semihydrogenation [i.e., C1–C2, 1.360(4) Å; C5–C6, 1.529(5) Å]. All other NMR and metrical data are very similar to that of the COD precursor, complex **1**. Complex **5** can also be formed by the slow (24 h) reaction of complex **1** with a large excess of COE, displacing COD. We have not determined the mechanism, but the absence of products that signal a dissociative process, such as a bridged COD complex, suggests that an associative process operates. Associative substitutions at d^8^ pincer complexes are well established.^21,44,45^

**Scheme 3:**
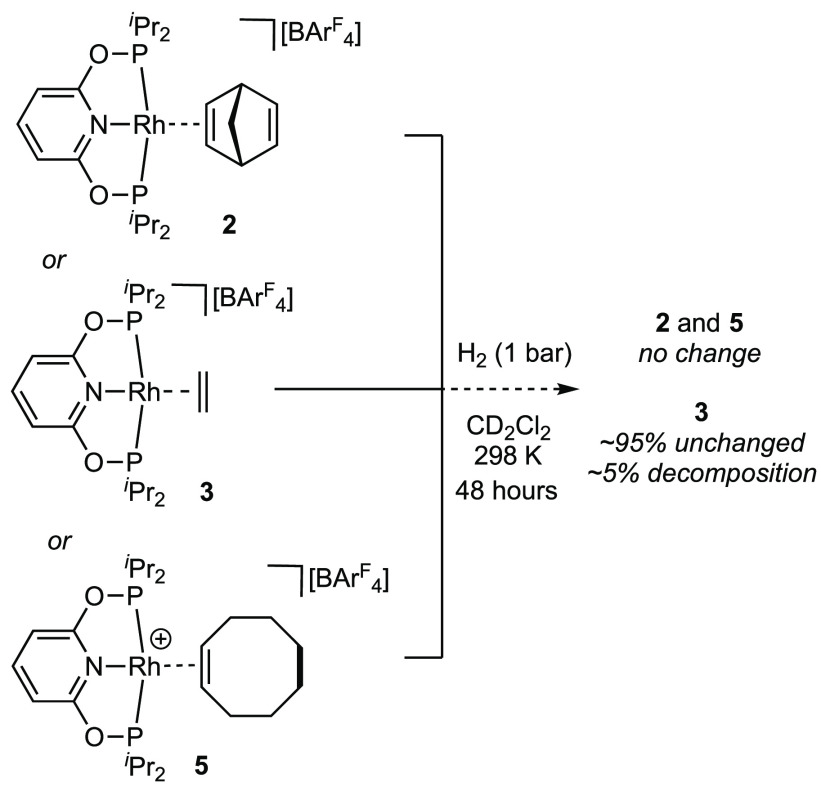
Attempted Reactivity of Complexes **2**, **3**, and **5** with H_2_

In contrast to complex **1**, exposure of the COE complex **5** to H_2_ (1 bar, CD_2_Cl_2_) over an extended period (48 h) returned complex **5** unchanged (Scheme 3). Identical behavior is observed for the NBD adduct **2**. For the ethene complex **3**, a small amount of decomposition (∼5%) to multiple unidentified hydride species is observed under these conditions. While minor, this decomposition signals very slow onward reactivity of **3** with H_2_ (vide infra). This overall attenuation of onward reactivity with H_2_ is in contrast to the faster (2 h) reaction of the COD complex **1**. The likely organometallic product of alkene hydrogenation, [Rh(PONOP-^i^Pr)(η^2^-H_2_)][BAr^F^_4_] **7**, based on previous studies on analogous [Rh(PONOP-^t^Bu)]^+^ systems,^17,18^ is not observed under these conditions (vide infra).

**Scheme 4:**
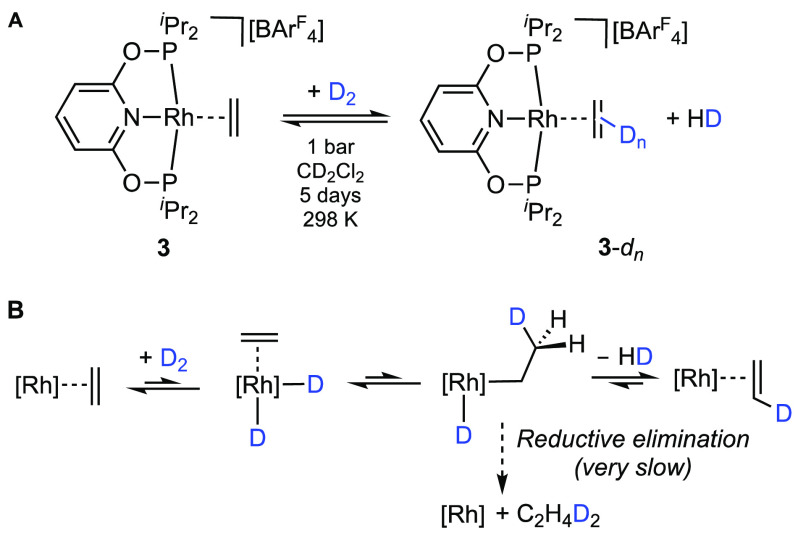
(A) Reaction of **3** with D_2_ and (B) Proposed Mechanism for H/D Exchange at Bound Ethene [Rh] = [Rh(PONOP-^i^Pr)]^+^.

Selective hydrogenation of the COD complex, but lack of observed reactivity of the NBD complex, is noteworthy because in cationic systems based upon [Rh(chelating diphosphine)(diene)]^+^ NBD is well established to undergo stoichiometric hydrogenation faster than COD.^46,47^ Informed by Goldman’s studies on the reactivity of Ir(carb–PNP)H_2_ with ethene,^14^ where reductive elimination from an intermediate ethyl hydride was found to be high in energy (Scheme 1) in the absence of external alkene, we studied the reactivity of the ethene complex **3** with D_2_ to probe the possibility of a similar situation occurring here (Scheme 4A). Over the course of 5 days, this resulted in the incorporation of a deuterium label into the bound ethene to give a mixture of isotopologues, **3**-*d*_*n*_. This was shown by ^1^H NMR spectroscopy (a ∼25% reduction in the relative integral of the η^2^-ethene resonance at δ 3.09 and a concomitant broadening due to H/D coupling), with a signal at δ 3.09 being observed in the ^2^H NMR spectrum and assigned to bound ethene, and electrospray ionization mass spectrometry (ESI-MS),^48^ which shows a mixture of isotopologues (**3**-*d*_*n*_, where *n* = 0–4; see the Supporting Information). These observations are consistent with the reversible oxidative addition of D_2_ to **3** and hydride migration to give an (unobserved) ethyl deuteride. Subsequent β-H transfer, followed by the reductive elimination of HD [observed, δ 4.55, t, *J*(HD) = 43 Hz], returns isotopically enriched **3**-*d* (Scheme 4B). Decomposition to a number of unidentified species also occurs over 5 days of exposure to D_2_, as noted for reactivity with H_2_.

**Scheme 5:**
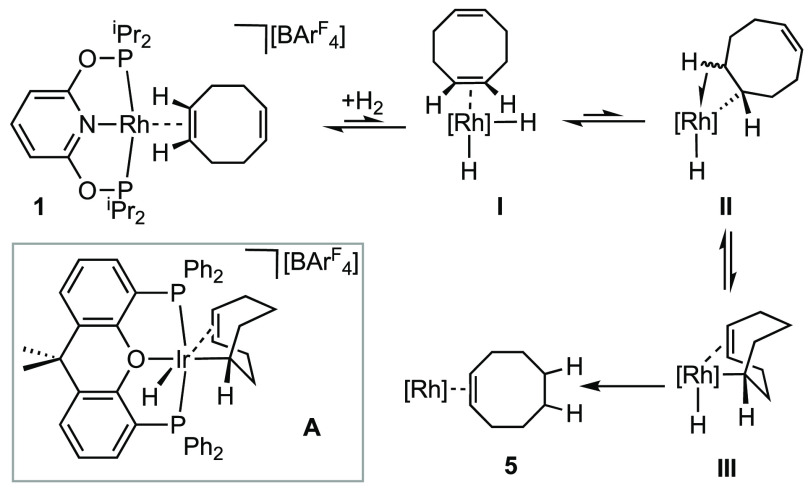
Proposed Mechanism for Intramolecular Alkene-Assisted Reductive Elimination in Complex **1** The inset shows the structure of a σ,π-C_8_H_13_ intermediate identified spectroscopically in the hydrogenation of **A**.^50^ [Rh] = [Rh(PONOP-^i^Pr)]^+^.

**Scheme 6:**
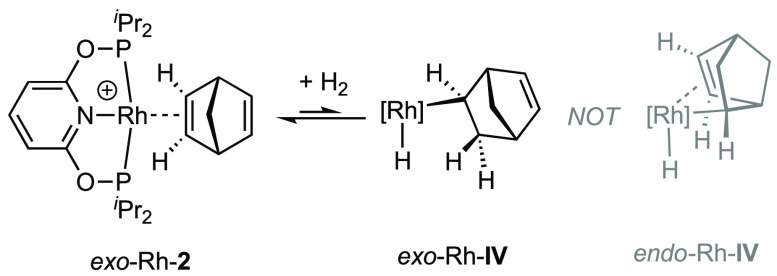
Exo Coordination of the Metal Fragment, Leading to Exo Metalation Rather Than Endo Metalation [Rh] = [Rh(PONOP-^i^Pr)]^+^.

We propose that, for the COD complex, where alkene hydrogenation is observed, reductive bond formation from the alkyl hydride intermediate is promoted by *intramolecular* coordination of the distal alkene. This is related to the Ir(carb–PNP)H_2_ system, where coordination of exogenous ethene acts to promote reductive elimination,^14^ or the similar role suggested for intramolecular C–H agostic interactions in H_2_ reductive elimination from Ir(P^t^Bu_2_Ph)_2_(CCPh)(H).^49^ A plausible reaction sequence is shown in Scheme 5. The key intermediate is **III**, which has a σ,π-C_8_H_13_ ligand in which the flexible σ-cycloctenyl ligand can engage in additional intramolecular alkene bonding. Complexes with such a ligand motif have been spectroscopically characterized as an intermediate in the closely related hydrogenation of [Ir(Xantphos)(η^2^η^2^-COD)][BAr^F^_4_] (**A**)^50^ and crystallographically characterized as Ir(σ,π-C_8_H_13_)(CO)_2_(AsPh_3_).^51^ This motif is unavailable for the ethene or COE complexes because of the lack of an additional alkene group. For the NBD complex **2**, geometric constraints must mean that such a σ,π-intermediate is not accessible. The initial exo coordination of the alkene (Figure 1) would result in an intermediate, *exo*-Rh-**IV** (Scheme 6) that is unable to partake in a σ,π-coordination mode, compared to *endo*-Rh-**IV** which we propose is not accessible. Exo addition of D_2_ to norbornene (NBE) analogues of **2** is well established.^52−54^ Attempts to promote this reactivity with another, suitable, intermolecular donor, acetonitrile, led to the simple displacement of the alkene and coordination of the nitrile, as measured by ^1^H and ^31^P{^1^H} NMR spectroscopy and ESI-MS.^55^ However, excess alkene (COD or ethene) does promote hydrogenation, as detailed in section 2.3.

This general lack of reactivity of the [Rh(PONOP-^i^Pr)(η^2^-alkene)][BAr^F^_4_] complexes described here with H_2_ is in contrast to the closely related complexes [Rh(PONOP-^t^Bu)(η^2^-alkene)][BAr^F^_4_] (L = ethene, η^2^-COD), which react rapidly with H_2_ to form the dihydrogen complex [Rh(PONOP-^t^Bu)(η^2^-H_2_)][BAr^F^_4_],^17,18^ with associated formation of the corresponding alkane. We suggest that this points to a different, dissociative, mechanism operating for ^t^Bu variants, consistent with the formation of η^2^η^2^-COD-bridged dimers,^18^ and previous observations on preferred coordination motifs in Ir(POCOP-R)-based systems (R = ^t^Bu, ^i^Pr).^19^

### 2.3 Catalysis

**Scheme 7:**
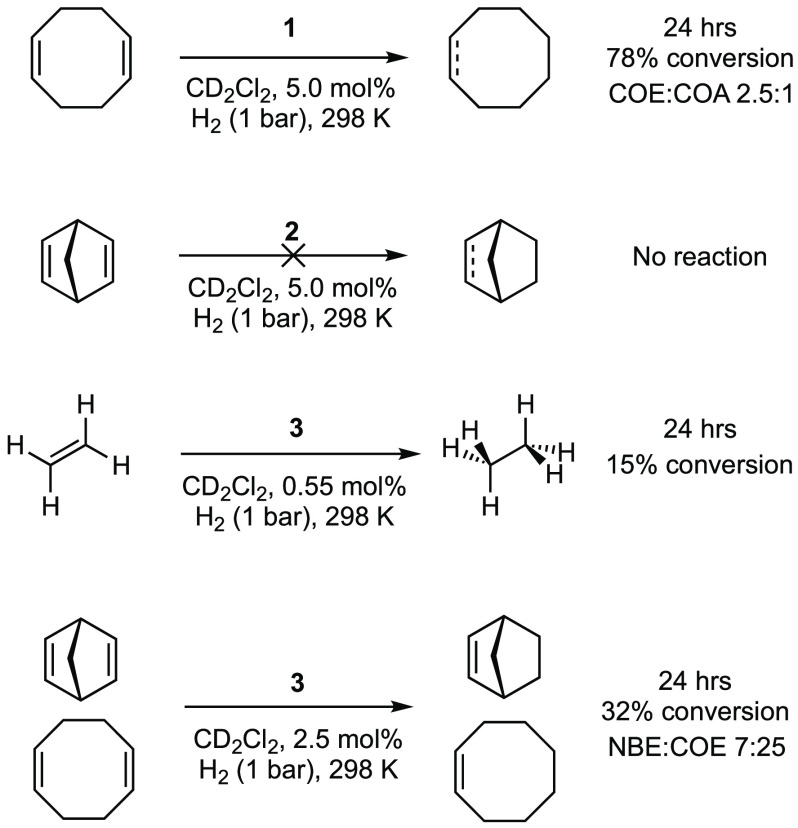
Catalytic Alkene Hydrogenation Reactions

While the monoalkene complexes do not undergo appreciable reaction with H_2_ on their own, in the presence of excess alkene catalytic turnover, albeit slow, can occur (Scheme 7). These are unoptimized conditions and simply demonstrate turnover rather than catalytic efficiency. For complex **1**, at 5 mol % after 24 h, 78% conversion of the COD substrate to give a mixture of COE and COA (2.5:1 ratio) is observed, with an overall turnover number (TON) ∼ 20. Using the ethene complex **3**, now at lower loadings (0.55 mol %), a similar TON for ethene hydrogenation is achieved after 24 h (TON = 27). For the NBD catalyst **2**, there is no detectable hydrogenation of NBD. These observations are fully consistent with the role of additional alkene in promoting hydrogenation. Now, rather than intramolecular assistance, as seen for complex **1**, the exogenous alkenes COD and ethene are promoters. With NBD as a substrate, we suggest that coordination of an additional NBD ligand at the metal center is disfavored because of its globular three-dimensional structure, and thus there is no overall hydrogenation. NBD *is* slowly hydrogenated to NBE in the presence of 1 equiv of COD per NBD using catalyst **3** (2.5 mol %). COD or COE can presumably coordinate to promote reductive elimination from a norbornyl intermediate, *exo*-Rh-**IV**. COE is also formed in this reaction from the competitive hydrogenation of COD.

### 2.4 Formation of the Dihydrogen Adduct **7**

**Scheme 8:**
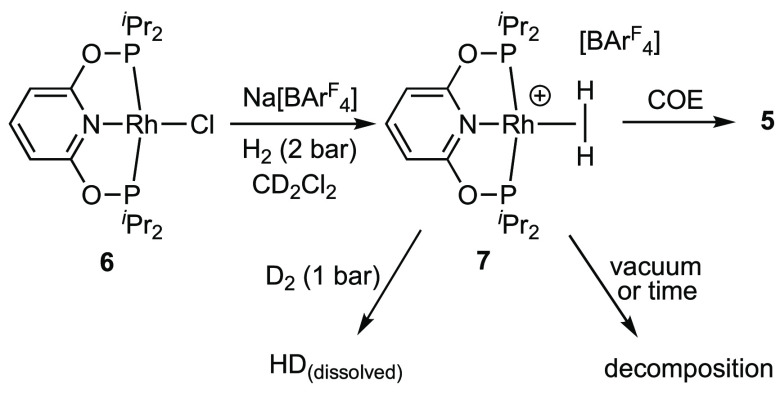
Synthesis of the Dihydrogen Complex **7**

The lack of onward reactivity of the monoalkene complexes with excess H_2_ under stoichiometric conditions might suggest that the expected product of such a reaction, **7**, is not accessible. We show that this is not the case. Using an in situ halide abstraction route,^17^ treatment of the neutral chloride complex Rh(PONOP-^i^Pr)Cl (**6**; see the Supporting Information) with Na[BAr^F^_4_] in a CD_2_Cl_2_ solution under 2 bar of H_2_ results in the formation of a new species in ∼80% purity that is spectroscopically characterized as the dihydrogen complex [Rh(PONOP-^i^Pr)(η^2^-H_2_)][BAr^F^_4_], **7** (Scheme 8). A precipitate, presumed to be NaCl, is also observed to be formed. Complex **7** is unstable in solution, decomposing at 298 K under a H_2_ atmosphere over 48 h to a mixture of products, likely those arising from reaction with the CD_2_Cl_2_ solvent under these conditions.^56^ In contrast, the ^t^Bu analogue is reported to be more stable and can be prepared directly from the ethene precursor.^17^ The independent synthesis of **7** suggests that the lack of reactivity of the monoalkene complexes with H_2_ is a kinetic phenomenon. The small amount of decomposition observed for the alkene complex **3** under a H_2_ atmosphere (ca. 5% after 48 h) may point to the slow formation of complex **7**, vide infra, which then decomposes at a rate faster than its formation.

Under these conditions, at 298 K, complex **7** is characterized by a broad, relative integral 2 H, whose signal is observed at δ –8.4 [full width at half-maximum (fwhm) = 150 Hz] in the ^1^H NMR spectrum. Dissolved H_2_ (δ 4.59) is also observed as a broad signal, suggesting exchange between free and bound H_2_ that is likely to be both temperature- and pressure-dependent. Upon cooling, both of these signals sharpen and move further apart in frequency, supporting such a process. At 235 K (500 MHz), a *T*_1_(min) of 48 ± 6 ms is measured on the signal now at δ −9.20 (fwhm = 90 Hz) that clearly identifies **7** as a dihydrogen complex. [Rh(PONOP-^t^Bu)(η^2^-H_2_)][BAr^F^_4_] shows similar spectroscopic data: δ −8.26 and *T*_1_(min) = 33 ms (264 K).^17^ Further evidence for slow exchange with free H_2_ in complex **7** comes from the removal of free H_2_ under vacuum, which causes the high-field dihydrogen signal in **7** to sharpen and reveal coupling to ^103^Rh: *J*(RhH) = 27.8 Hz. However, this removal of the H_2_ atmosphere also results in the decomposition of complex **7** to a range of products (50% decomposition in 20 min), an observation that supports the H_2_ ligand being rather labile. Rapid freeze–pump–thawing of a CD_2_Cl_2_ solution of **7** and then the addition of excess COE reforms complex **5** almost quantitively. When **7** is exposed to a D_2_ atmosphere (10 min, 1 bar), HD(dissolved) is observed as a broad 1:1:1 triplet, δ 4.41 [*J*(DH) = 43 Hz, measured at 255 K], showing that bond metathesis between D_2_ and bound H_2_ can occur that likely involves a dideuterium/dihydride intermediate, [Rh(PONOP-^i^Pr)(η^2^-D_2_)(H)_2_][BAr^F^_4_], operating via a σ-CAM mechanism.^57^ That this dihydrogen complex **7** is not observed when H_2_ is added to the alkene complexes, but there is H/D exchange into the bound ethene complex **3**, indicates that reductive elimination of an alkyl hydride is the rate-limiting step, but this intermediate must be in endergonic equilibrium with the observed starting complex **3**, i.e., Scheme 4. Interested in probing this further, we turned to the use of *p*H_2_ to help identify any intermediates in this process.^58^

### 2.5 Observation of the Dihydrogen Adduct under Catalytically Relevant Conditions Using *p*H_2_ via the PNL Effect

**Figure 3:**
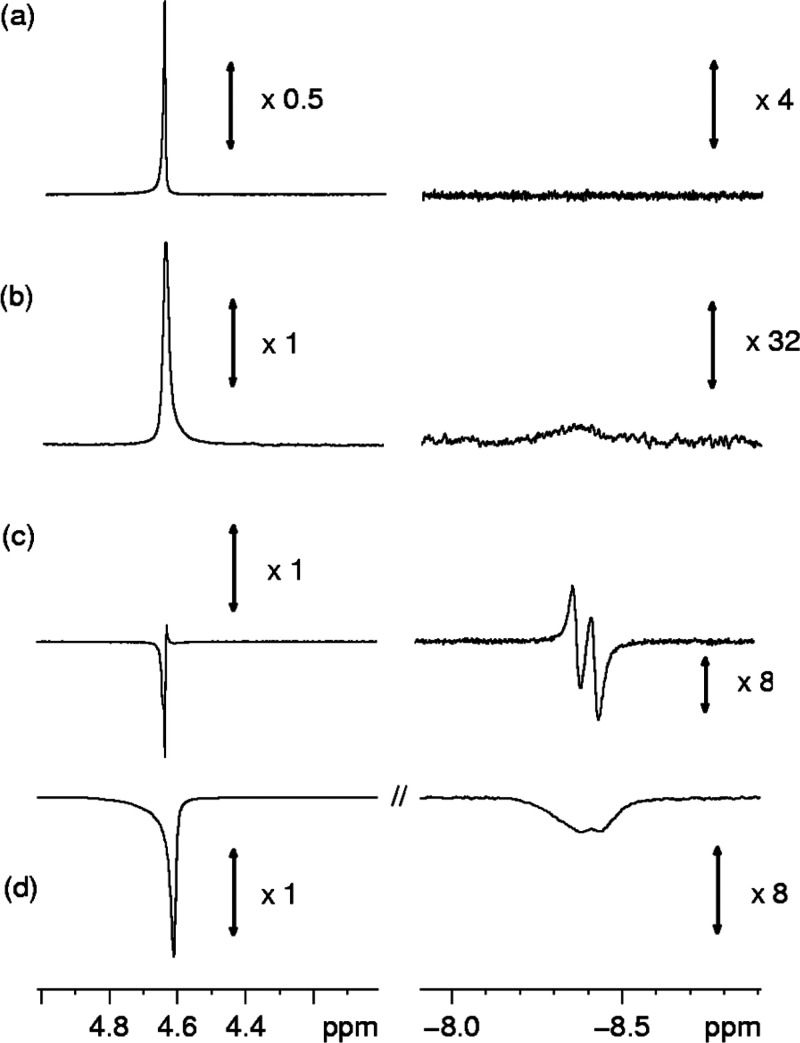
Selected regions of a series of single-scan (unless stated) ^1^H NMR spectra recorded at 500 MHz during monitoring of the reaction of **3** with *p*H_2_. The corresponding vertical expansion settings are indicated. (a) Thermally polarized result, 32 scans, 298 K. (b) Thermally polarized single scan after shaking of the tube, 248 K. (c) ^31^P decoupled with a 45° observation pulse single scan, 238 K, and (d) with a 90° observation pulse, and no ^31^P decoupling, single scan, 238 K.

When **3** is exposed to *p*H_2_ at 298 K (3 bar) in a 5 mm J. Young NMR tube, ^1^H NMR observation at 298 K reveals a broad H_2_ signal at δ 4.6 (Figure 3a), which exhibits a PNL effect^15,59^ when additionally observed with a 45° pulse. A relatively strong H_2_ peak can also be observed at this position through the observe *para*hydrogen only spectroscopy (OPSY^60^) sequence that selectively detects two spin-order terms while filtering out the thermal single-spin components. These two observations together indicate the presence of slow exchange between free and a rhodium-bound H_2_ species. At this early temporal stage in the reaction, this is consistent with the reversible formation of [Rh(PONOP-^i^Pr)(η^2^-C_2_H_4_)(η^2^-H_2_)][BAr^F^_4_] from complex **3** and/or its dihydride isomer [Rh(PONOP-^i^Pr)(η^2^-C_2_H_4_)(H)_2_][BAr^F^_4_] (e.g., Scheme 4b).^61^ No complex **7** is observed that this point.

Repeating these measurements, at 3 bar of *p*H_2_, but then subsequent removal of the sample from the magnet, shaking under ambient laboratory conditions and return, resulted in the additional observation, at very low concentration, of transient **7**, as indicated by the appearance of a broad signal at δ −8.3 (Figure 3b) at 248 K. This signal sharpens partially into a doublet of ∼24 Hz under ^31^P decoupling conditions. Upon further cooling to 238 K, the size of the PNL signal of free H_2_ increases, and, remarkably, after the application of a 45° pulse under ^31^P decoupling conditions, the original signal at δ −8.3 that is assigned to **7** is now also observed as two antiphase signals, with frequency separations of 25 and 10.4 Hz^62^ (Figure 3c). The former is consistent with a *J*(RhH) splitting. This process of removal of the sample from the spectrometer, shaking, and return to observe both the antiphase free H_2_ and high-field signals can be repeated a number of times. These data confirm that there is a substantial signal gain associated with these resonances from a PHIP effect (*p*-hydrogen-induced polarization), and we thus conclude that a metal complex of a dihydrogen ligand exhibits a clear PNL effect in a manner directly analogous to that of the free H_2_ signal. For this PHIP-enhanced dihydrogen complex **7**, the high-field signal in the ^31^P-*coupled*^1^H NMR spectrum exhibits the same intrinsic behavior but is now observed to be much broader, suffering from signal cancellation.^63^ The detection of such character is indicative of the observation of a two-spin-order term, like that associated with the PASADENA^58^ detection of an AX spin system formed by *p*H_2_ addition. This leads to the creation of a longitudinal two-spin-order term that reflects the involvement of both *p*H_2_-derived proton spins and results in a flip-angle dependence to the observed response: zero with a 90° excitation pulse and maximized for the 45° pulse. We tested this hypothesis, and, as expected, when the same measurement was completed with a 90° excitation pulse, the two signals were found to lose their positive peak contributions but, rather than disappear, remained with negative line intensity (Figure 3d). This suggests that the competitive creation of net single-spin polarization in H_2_ also occurs as a result of differential relaxation experienced by the two protons in what was *p*H_2_ after binding and prior to the re-formation of free H_2_, as described by Aime under ALTADENA conditions.^64^ The resulting single spin order is then visible through the action of the 90° excitation pulse in the normal way, although it appears with negative amplitude relative to the thermal Zeeman-derived magnetization.

**Scheme 9:**
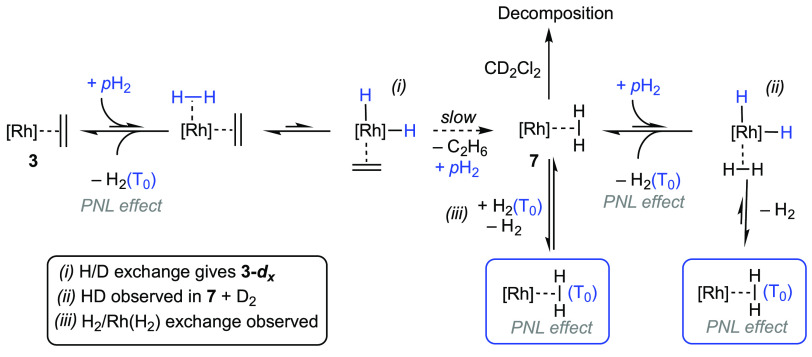
Suggested Pathways for the Generation of Triplet H_2_ [H_2_(T_0_)] and Its Incorporation in the Dihydrogen Complex **7** [Rh] = [Rh(PONOP-^i^Pr)][BAr^F^_4_].

The *p*H_2_-enhanced NMR signal seen for both free and, remarkably, bound H_2_ in this study reflect unusual, but theoretically predicted, behavior for a system that is under slow exchange.^15^*p*H_2_ is simply NMR-silent molecular H_2_ with singlet nuclear spin order. Three other spin orders that are associated with H_2_(T_0_) are possible, also known as *o*H_2_, and are NMR-active. Normally, the NMR signal-intensity-enhancing effect PHIP is associated with the observation of stable pairwise H_2_ oxidative addition products with magnetically distinct hydride ligands.^65,66^ However, more complex behavior reflective of the one-proton PHIP effect,^67^ and more recently the PNL effect,^15^ can occur. The latter effect leads to the observation of a negative signal for dissolved molecular hydrogen and has been explained by singlet–triplet state conversion caused by exchange between H_2_ and a transient dihydrogen complex. Such a scenario is shown in Scheme 9, in which there are a number of steps in the reaction manifold that forms complex **7** that can also account for the generation of H_2_(T_0_), as supported by the H/D exchange experiments already described. In the specific case here, an additional exchange between the dissolved H_2_(T_0_) and bound, but labile, H_2_ in **7** can also lead to the remarkable signal enhancement seen in the high-field Rh(η^2^-H_2_) signal of **7**. Our observations also show that while **7** is formed slowly upon hydrogenation of **3**, because it also exchanges very rapidly at 298 K with free H_2_, we fail to see an enhanced signal at this temperature, although it is visible under standard conditions. This is compounded by the decomposition of **7** at 298 K, which is competitive with its slow formation.

## 3 Conclusions

While hydrogenation of the simple alkene adducts of {Rh(pincer)(alkene)} is an attractive and expedient methodology to remove the alkene and generate a reactive dihydride/dihydrogen complex, our observations here indicate that the identity of the alkene in such complexes with the PONOP-^i^Pr ligand can be crucial in onward reactivity and thus needs to be considered in complex or catalyst design. The lack of reactivity of the monoalkene complexes with H_2_ is in contrast with the bulkier ^t^Bu analogues, which react readily to form the corresponding dihydrogen complexes. Such observations are related, in a more general sense, to [Rh(phosphine)(diene)]^+^ complexes, which are important precatalysts for a wide variety of transformations, and often activated by hydrogenation of the bound alkene ligand, the identity of which can also be important.^5,46,52^ Moreover, the specific reactivity that the [Rh(PONOP-^i^Pr)(η^2^-ethene)]^+^ complex presents (i.e., reversible H_2_ addition and the slow formation of a dihydrogen complex that undergoes exchange with dissolved H_2_) leads to an unusual situation where a signal enhancement due to the PNL effect^15^ in the bound dihydrogen ligand can be observed as a consequence of the initial addition of *p*H_2_. While the original PHIP effect has been known since 1987^58^ and is used widely to detect dihydride oxidative addition products at transition metal centers, to our knowledge, this is the first example where a dihydrogen ligand has been directly observed as an enhanced signal using *p*H_2_. While the precise reaction manifold that leads to this is still to be determined, the observation of this effect is noteworthy in itself and encourages further investigation.

## Supporting Information Available

Full experimental details, characterization data, and details of single-crystal X-ray diffraction experiments (PDF)

## Accession Codes

CCDC 2050110–2050114 contain the supplementary crystallographic data for this paper. These data can be obtained free of charge via www.ccdc.cam.ac.uk/data_request/cif, or by emailing data_request@ccdc.cam.ac.uk, or by contacting The Cambridge Crystallographic Data Centre, 12 Union Road, Cambridge CB2 1EZ, UK; fax: +44 1223 336033.
