# Supplementary material for: η2-Alkene Complexes of [Rh(PONOP-iPr)(L)]+ Cations (L = COD, NBD, Ethene). Intramolecular Alkene-Assisted Hydrogenation and Dihydrogen Complex [Rh(PONOP-iPr)(η-H2)]+
Source: Inorg Chem. 2021 Feb 11;60(18):13903–12. doi: 10.1021/acs.inorgchem.0c03687 (PMC8456414; doi:10.1021/acs.inorgchem.0c03687)
Supplement: Supplementary file 1 — ic0c03687_si_001.pdf [file ic0c03687_si_001.pdf]

Electronic Supporting Information (ESI)

η^2-Alkene Complexes of [Rh(PONOP-^{i}Pr)(L)]$^+$ Cations (L = COD, NBD, ethene); Intramolecular Alkene-Assisted Hydrogenation and a Dihydrogen Complex [Rh(PONOP-^{i}Pr)(η-H_2)]$^+$

Alice Johnson,[a†] Cameron G. Royle,[a,b] Claire N. Brodie,[b] Antonio J. Martínez-Martínez,[a‡] Simon B. Duckett,[b] Andrew S. Weller[b*]

[a] Department of Chemistry, Chemistry Research Laboratories, University of Oxford, Mansfield Road, Oxford, OX1 3TA, United Kingdom.

[b] Department of Chemistry, University of York, Heslington, York, YO10 5DD, United Kingdom.

† Present address: Department of Chemistry, University of Leicester, University Road, Leicester, LE1 7RH, United Kingdom.

‡ Present address: CISQO – Centre for Research in Sustainable Chemistry, CISQO, University of Huelva, 21007 Huelva, Spain.

* E-mail: andrew.weller@york.ac.uk

Table of Contents

1. Experimental 3

1.1. General Procedures 3

1.2. Syntheses 4

1.3. Crystallography 13

1.4. Angles between planes – alkene complexes 16

1.5. NMR Spectra for iPr-PONOP (L1) 17

1.6. NMR Spectra for 1 18

1.7. NMR Spectra for 2 19

1.8. NMR Spectra for 3 20

1.9. NMR Spectra for 4 21

1.10. NMR Spectra for 5 22

1.11. NMR Spectra for 6 23

1.12. NMR Spectra for 7 under an H_2 atmosphere 24

1.13. NMR Spectra for 7 in the absence of an H_2 atmosphere 25

1.14. Variable Temperature ^{31}P NMR Complex 4 26

1.15. Variable Temperature ^{1}H NMR Complex 7 ... 27

1.16. Variable Temperature ^{1}H NMR Spectra for the reaction of Complex 7 with D_2 ... 28

1.17. Variable Temperature ^{2}H NMR Spectra for the reaction of Complex 7 with D_2 ... 29

1.18. Hydrogenation of Complex 1 ... 30

1.19. Catalytic Hydrogenation of COD with Complex 1 as Precatalyst ... 31

1.20. Catalytic Hydrogenation of COE with Complex 3 as Precatalyst ... 32

1.21. Catalytic Hydrogenation of C_2H_4 with Complex 5 as Precatalyst ... 33

1.22. Electrospray Mass Spectrometry; Comparison of Complexes 3 and 3-d_n ... 34

2. References ... 35

1. Experimental

1.1. General Procedures

All manipulations, unless otherwise stated, were performed under an argon atmosphere using standard Schlenk line and glove-box techniques. Glassware was oven-dried at 130 °C overnight and flamed under vacuum prior to use. CH_2Cl_2 and pentane were dried using a Grubbs-type solvent purification system (MBraun SPS-800) and degassed by three successive freeze-pump-thaw cycles. CD_2Cl_2 and 1,2-$C_6H_4F_2$ (pre-treated with alumina) were dried over CaH_2, vacuum distilled and stored over 3 Å molecular sieves. Na[$BArF_4$], [Rh(NBD)$_2$][$BAr^F{}_4$], and [Rh(COD)$_2$][$BAr^F{}_4$] were prepared by literature methods.[1,2] $RhCl_3{\cdot}(H_2O)_n$ was purchased from Precious Metals Online PMO Pty Ltd and used as received. Neat norbornadiene (NBD) was purchased from Sigma-Aldrich, distilled over freshly cut small pieces of sodium metal before subsequent three-fold freeze-pump thawing. D_2 gas was purchased from Sigma-Aldrich and used as received.

NMR spectra were recorded on a Bruker Avance III 500 MHz NMR spectrometer or a Bruker Avance III HD nanobay 400 MHz NMR spectrometer at room temperature. Residual *protio*-solvent was used as reference for ^{1}H spectra in deuterated solvent samples. $^{31}P\{^{1}H\}$ NMR spectra were externally referenced to 85% H_3PO_4. ^{1}H assignments were aided by $^{1}H\{^{31}P\}$ experiments. All chemical shifts (δ) are quoted in ppm and coupling constants (J) in Hz. ESI-MS were recorded by Karl Heaton at the University of York on a Bruker compact® time-of-flight mass spectrometer coupled to an Agilent 1260 Infinity series LC system. Elemental microanalyses were performed by Stephen Boyer at London Metropolitan University.

1.2. Syntheses

Synthesis of **iPr-PONOP** (**L1**)

This ligand was prepared by a modification of the literature procedure.[3]

In a 500 ml Rotaflo under argon was added 2,6-dihydroxypyridine hydrochloride (737.8 mg, 5 mmol), N,N,N',N'-tetramethylethylenediamine (10 mmol, 1.50 mL), and triethylamine (30.5 mmol, 4.25 mL) in 50 mL of dry THF. The reaction mixture was cooled to 0 °C, and a solution of $^{i}Pr_2PCl$ (11 mmol, 1.74 ml) in 10 mL of dry THF was added. After the mixture reached room temperature, it was heated to reflux with stirring for 5 days. The reaction mixture was cooled, filtered by cannula and solvent removed under reduced pressure to give the product as a colourless oil (1.6 g, 92%).

^{1}H NMR (400 MHz, C_6D_6) δ 7.04 (t, $^{3}J_{HH}$ = 7.8 Hz, 1H, Py), 6.36 (d, $^{3}J_{HH}$ = 7.8 Hz, 2H, Py), 1.98 (heptd, $^{3}J_{HH}$ = 7.1 Hz, $^{2}J_{PH}$ = 1.9 Hz, 4H, P-C*H*(CH_3)$_2$), 1.21 (dd, $^{3}J_{HP}$ = 10.8 Hz, $^{3}J_{HH}$ = 7.0 Hz, 12H, P-CH(C*H*$_3$)$_2$), 1.09 (dd, $^{3}J_{HP}$ = 14.9 Hz, $^{3}J_{HH}$ = 7.2 Hz, 12H, P-CH(C*H*$_3$)$_2$).

^{31}P NMR (162 MHz, C_6D_6) δ 145.96 (s).

Synthesis of [(^{i}Pr-PONOP)Rh(COD)][BAr$^F_4$] (**1**)

To a solution of [Rh(COD)$_2$][BAr$^F_4$] (118.2 mg, 0.1 mmol) in 1,2-difluorobenzene (10 ml) was added **L1** (1.0 ml, 0.1 M soln, 0.1 mmol) and the solution stirred for 1 h. The solution was filtered by cannula, concentrated under reduced pressure to approx. 1 ml, and pentane (10 ml) added to precipitate a yellow solid which was collected and vacuum dried to give the product (103.9 mg, 73%). Crystals of suitable quality for single-crystal X-ray diffraction studies were grown from a 1,2-difluorobenzene solution layered with pentane at 298 K.

^{1}H NMR (400 MHz, CD_2Cl_2) δ 7.83 (t, $^3J_{HH}$ = 8.1 Hz, 1H, Py), 7.72 (s, 8H, BAr$^F_4$), 7.56 (s, 4H, BAr$^F_4$), 6.79 (d, $^3J_{HH}$ = 8.1 Hz, 2H, Py), 5.64 (t, $^3J_{HH}$ = 3.7 Hz, 2H, COD unbound alkene CH), 4.83 (s, 2H, COD bound alkene CH), 2.59 – 2.47 (m, 4H, COD allylic C*H*), 2.42 (virt. heptet, $^3J_{HH}$ = 7.2 Hz, 4H, C*H*(CH_3)$_2$), 2.27 – 2.00 (m, 4H, COD), 1.29 (overlapping doublets, $^3J_{HH}$ = 7.2 Hz, 24H, CH(CH_3)$_2$).

^{31}P NMR (162 MHz, CD_2Cl_2) δ 200.17 (d, $^1J_{PRh}$ = 137.3 Hz).

Elem. anal. Calcd for $C_{57}H_{55}BF_{24}NO_2P_2Rh$: C, 48.29; H, 3.91; N 0.99. Found: C, 48.25; H, 3.99; N, 1.05.

ESI-MS (CH_2Cl_2) m/z found (calculated) for $C_{25}H_{43}NO_2P_2Rh$ [M]$^+$: 554.1819 (554.1824)

Molecular Structure:

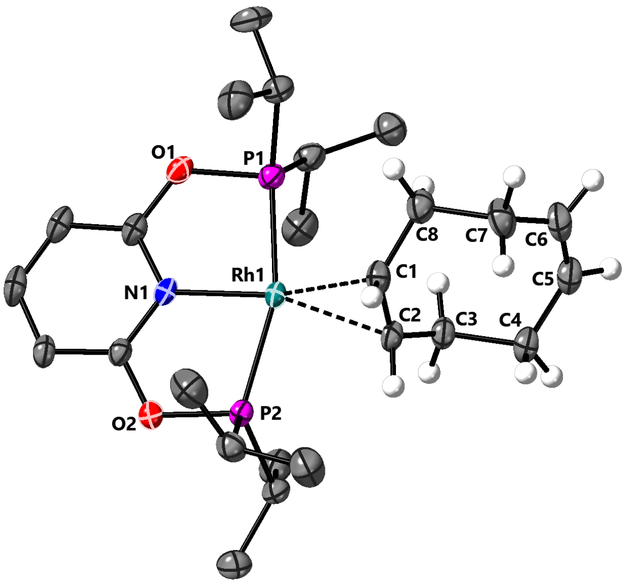

Figure S1: Molecular structure of **1** determined by single crystal X-ray diffraction. 50% displacement ellipsoids. Hydrogen atoms (except crystallographically-located COD moiety positions) and BAr$^F_4$ anion are omitted for clarity. Selected bond lengths [Å] and angles [°]: Rh(1)-P(1) 2.2908(12), Rh(1)-P(2) 2.2494(11), Rh(1)-N(1) 2.050(3), Rh(1)-C(1) 2.208(4), Rh(1)-C(2) 2.222(4), P(1)-O(1) 1.690(3), P(2)-O(2) 1.668(3), C(1)-C(2) 1.384(7),C(1)-C(8) 1.474(7), C(2)-C(3) 1.483(7), C(3)-C(4) 1.560(7), C(4)-C(5) 1.467(8), C(5)-C(6) 1.309(9), C(6)-C(7) 1.497(9), C(7)-C(8) 1.566(7), P(2)-Rh(1)-P(1) 160.20(4), N(1)-Rh(1)-P(1) 80.26(10), N(1)-Rh(1)-P(2) 80.34(10), N(1)-Rh(1)-C(1) 161.07(16), N(1)-Rh(1)-C(2) 160.64(16).

Synthesis of [(^{i}Pr-PONOP)Rh(NBD)][BAr$^F{}_4$] (**2**)

To a solution of $[Rh(NBD)_2][BAr^F{}_4]$ (115.0 mg, 0.1 mmol) in 1,2-difluorobenzene (10 ml) was added **L1** (1.0 ml, 0.1 M soln, 0.1 mmol) and the solution stirred for 1 h. The solution was filtered by cannula, concentrated under reduced pressure to approx. 1 ml, and pentane (10 ml) added to precipitate a yellow solid which was collected and vacuum dried to give the product (93.2 mg, 67%). Crystals of suitable quality for single-crystal X-ray diffraction studies were grown from a 1,2-difluorobenzene solution layered with pentane at 298 K.

^{1}H NMR (400 MHz, CD_2Cl_2) δ 7.79 (t, $^3J_{HH}$ = 8.1 Hz, 1H, Py), 7.72 (s, 8H, $BAr^F{}_4$), 7.56 (s, 4H, $BAr^F{}_4$), 6.82 (d, $^3J_{HH}$ = 8.1 Hz, 2H, Py), 6.58 (s, 2H, NBD unbound alkene-CH), 4.33 (tm, $^3J_{HH}$ = 8 Hz, 2H, Rh bound alkene-CH), 3.44 (s, 2H, NBD bridgehead), 2.77 (virtual hept, $^3J_{HH}$ = 7 Hz, 4H, CH(CH$_3$)$_2$), 1.72 (dm, $^2J_{HH}$ = 7.5 Hz, 1H, NBD bridge-CH), 1.32 (overlapping doublets, $^3J_{HH}$ = 7.1 Hz, 24H, CH(CH_3)$_2$), 1.22 (dm, $^2J_{HH}$ = 7.5 Hz, 1H, NBD bridge-CH).

^{31}P NMR (162 MHz, CD_2Cl_2) δ 211.44 (d, $^1J_{PRh}$ = 124.5 Hz).

Elem. anal. Calcd for $C_{56}H_{51}BF_{24}NO_2P_2Rh$: C, 47.99; H, 3.67; N 1.00. Found: C, 47.78; H, 3.83; N, 1.04.

ESI-MS (CH_2Cl_2) m/z found (calculated) for $C_{25}H_{45}NO_2P_2Rh$ $[M]^+$: 537.1428 (537.1433).

Molecular Structure:

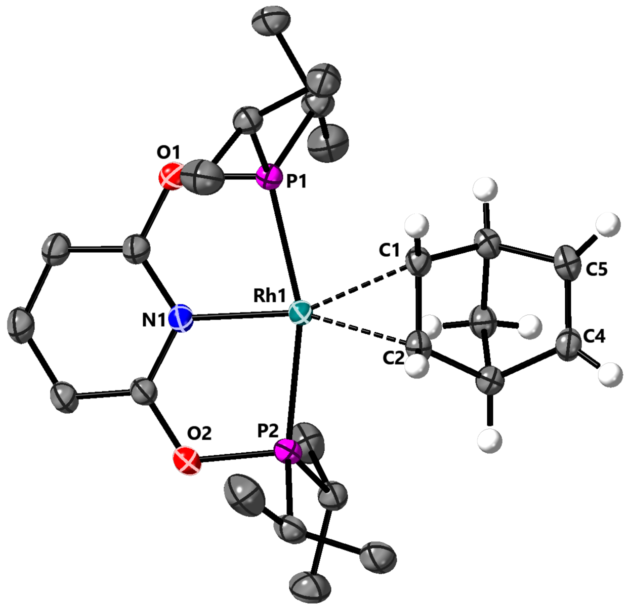

Figure S2: Molecular structure of **2** determined by single crystal X-ray diffraction. One of four independent cationic fragments located in the unit cell is presented. 50% displacement ellipsoids. Hydrogen atoms (except crystallographically-located NBD moiety positions) and $BAr^F{}_4$ anion are omitted for clarity. Selected bond lengths [Å] and angles [°]: Rh(1)-P(1) 2.2522(8), Rh(1)-P(2) 2.2712(8), Rh(1)-N(1) 2.053(3), Rh(1)-C(1) 2.188(3), Rh(1)-C(2) 2.175(3), P(1)-O(1) 1.674(3), P(2)-O(2) 1.678(3), C(1)-C(2) 1.391(5), C(4)-C(5) 1.329(6), P(1)-Rh(1)-P(2) 160.02(3), N(1)-Rh(1)-P(1) 80.24(8), N(1)-Rh(1)-P(2) 79.83(8), N(1)-Rh(1)-C(1) 160.39(12), N(1)-Rh(1)-C(2) 161.82(12).

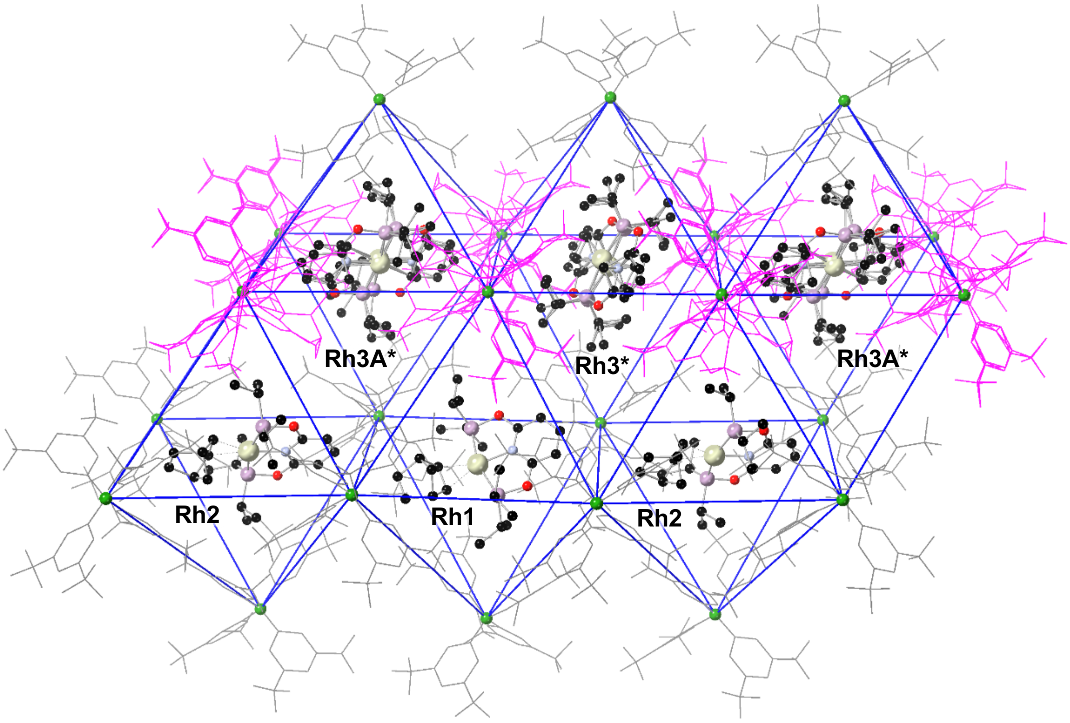

Figure S3: Extended structure of **2** as determined by single-crystal X-ray diffraction, demonstrating the solid-state packing pattern of bicapped parallelepipedal motifs (10 BAr^F_4 anions about two cationic fragments). Cationic fragments are represented as ball-and-stick with rhodium atoms identified (*note the disordered Rh3 and Rh3A fragments, where two 0.5 chemical occupancy fragments are disordered over the same position). BAr^F_4 anions are represented as wireframes with B atom balls. Pairs of 0.5 chemical occupancy BAr^F_4 anions disordered over the same position are highlighted in magenta.

Synthesis of [(^{i}Pr-PONOP)Rh(C_2H_4)][BAr$^F{}_4$] (**3**)

To a solution of **4** (131.4 mg, 0.05 mmol) in 1,2-difluorobenzene (10 ml) was added [Rh(C_2H_4)$_2$Cl]$_2$ (19.4 mg, 0.05 mmol) and the mixture stirred in the dark for 2 h. The mixture was filtered by cannula to give a yellow solution which was concentrated under reduced pressure to approx. 1 ml and pentane (10 ml) added to precipitate a yellow/orange solid which was collected and vacuum dried to give the product (100.2 mg, 75%). Crystals of suitable quality for single-crystal X-ray diffraction studies were grown from a 1,2-difluorobenzene solution layered with pentane at 298 K.

^{1}H NMR (400 MHz, CD_2Cl_2) δ 7.84 (t, $^3J_{HH}$ = 8.1 Hz, 1H, Py), 7.72 (s, 8H, BAr$^F{}_4$), 7.56 (s, 4H, BAr$^F{}_4$), 6.87 (d, $^3J_{HH}$ = 8.1 Hz, 2H, Py), 3.09 (td, $^3J_{HP}$ = 3.3 Hz, $^2J_{HRh}$ 1.7 Hz, 4H, C_2H_4), 2.62 (virtual hept, $^3J_{HH}$ = 7.1 Hz, 4H, C*H*(CH_3)$_2$), 1.24 (m, 24H, CH(C*H*$_3$)$_2$).

^{31}P NMR (162 MHz, CD_2Cl_2) δ 213.35 (d, $^1J_{PRh}$ = 123.7 Hz).

Elem. anal. Calcd for $C_{51}H_{47}BF_{24}NO_2P_2Rh$: C, 45.80; H, 3.54; N 1.05. Found: C, 45.78; H, 3.53; N, 1.04.

ESI-MS (CH_2Cl_2) m/z found (calculated) for $C_{19}H_{35}NO_2P_2Rh$ [M]$^+$: 474.1129 (474.1153) (note: the similar [(*PONOP*-^{i}Pr)Rh(N_2)]$^+$, which might conceivably be observed under the conditions of mass spectrometry, is calculated at m/z = 474.0941).

Molecular Structure:

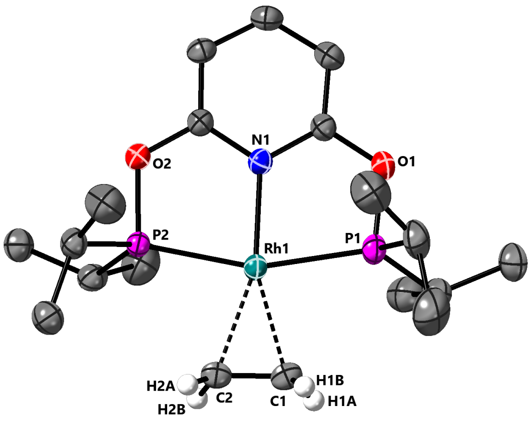

Figure S4: Molecular structure of **5** determined by single crystal X-ray diffraction. 50% displacement ellipsoids. Hydrogen atoms (except crystallographically-located C_2H_4 positions) and BAr$^F{}_4$ anion are omitted for clarity. Selected bond lengths [Å] and angles [°]: Rh(1)-P(1) 2.2642(10), Rh(1)-P(2) 2.2603(9), Rh(1)-N(1) 2.038(3), Rh(1)-C(1) 2.165(5), Rh(1)-C(2) 2.170(5), P(1)-O(1) 1.672(3), P(2)-O(2) 1.675(3), C(1)-C(2) 1.326(8), P(2)-Rh(1)-P(1) 160.27(4), N(1)-Rh(1)-P(1) 80.18(9), N(1)-Rh(1)-P(2) 80.27(9), N(1)-Rh(1)-C(1) 161.72(18), N(1)-Rh(1)-C(2) 161.86(18).

Synthesis of [{(^{i}Pr-PONOP)Ag}$_2$][BAr$^F{}_4$]$_2$ (**4**)

To a solution of [Ag(NCMe)$_2$][BAr$^F{}_4$] (105.3 mg, 0.1 mmol) in CH_2Cl_2 (10 mL) was added **L1** (1.0 ml, 0.1 M soln, 0.1 mmol) and the solution stirred for 1 h in the dark. The solution was filtered through celite, the filtrate concentrated to approx. 1 mL under reduced pressure and pentane (10 ml) added to precipitate a white solid which was collected and vacuum dried to give the product (123.5 mg, 94%). Crystals of suitable quality for single-crystal X-ray diffraction studies were grown from a CH_2Cl_2 solution layered with pentane and with complete exclusion of light at 298 K.

^{1}H NMR (400 MHz, CD_2Cl_2) δ 7.79 (t, $^3J_{HH}$ = 7.9 Hz, 1H, Py), 7.72 (s, 8H, BAr$^F{}_4$), 7.56 (s, 4H, BAr$^F{}_4$), 6.70 (d, $^3J_{HH}$ = 7.9 Hz, 2H, Py), 2.38 (hept, $^3J_{HH}$ = 6.5 Hz, 4H, C*H*(CH$_3$)$_2$), 1.31 – 1.17 (m, 24H, CH(C*H$_3$*)$_2$).

^{31}P NMR (162 MHz, CD_2Cl_2) δ 135.24 (d, $^1J_{AgP}$ = 548.7 Hz).

Elem. anal. Calcd for $C_{98}H_{86}Ag_2B_2F_{48}N_2O_4P_4$: C, 44.77; H, 3.30; N 1.07. Found: C, 44.63; H, 3.19; N, 1.18.

Molecular Structure:

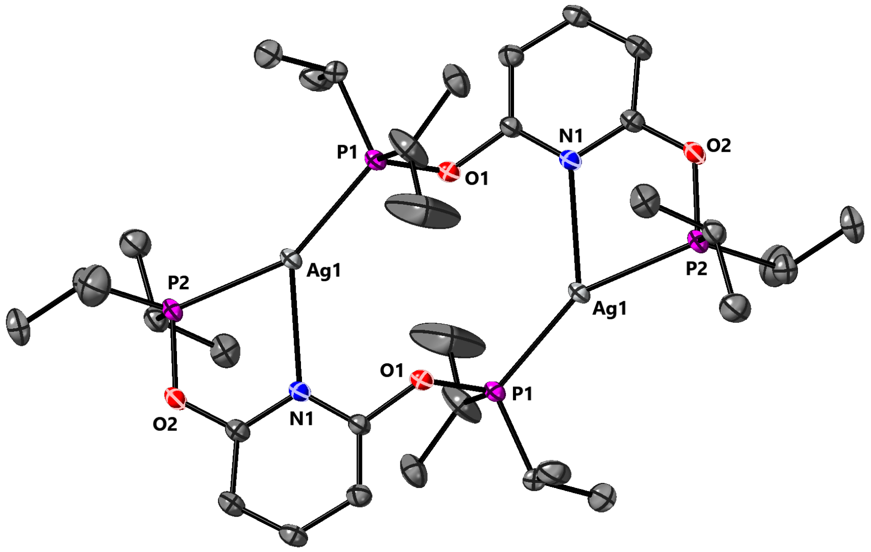

Figure S5: Molecular structure of **4** determined by single crystal X-ray diffraction. 50% displacement ellipsoids. Hydrogen atoms and BAr$^F{}_4$ anion are omitted for clarity. Selected bond lengths [Å] and angles [°]: Ag(1)-P(1) 2.3691(6), Ag(1)-P(2)#1 2.4208(6), Ag(1)-N(1)#1 2.428(2), P(1)-O(1) 1.6384(19), P(2)-O(2) 1.6583(19), P(1)-Ag(1)-P(2)#1 153.53(2), P(1)-Ag(1)-N(1)#1 132.36(5), P(2)#1-Ag(1)-N(1)#1 74.07(5).

Synthesis of [(^{i}Pr-PONOP)Rh(COE)][BAr$^F_4$] (**5**)

In a Young's flask, a solution of **1** (25 mg) in CH_2Cl_2 (2.5 ml) was placed under an atmosphere of H_2 (1 atm, freeze-pump-thaw cycles) and the solution stirred. After 2 h, the solution was frozen with liquid N_2, exposed to dynamic vacuum ($p < 10^{-2}$ mbar) and repressurised under argon once thawed. The mixture was concentrated under reduced pressure to approx. 1 ml and pentane (10 ml) added to precipitate a yellow/orange solid which was collected and vacuum dried to give the product (20 mg, 80%).

^{1}H NMR (400 MHz, CD_2Cl_2) δ 7.82 (t, $^3J_{HH}$ = 8.1 Hz, 1H, Py), 7.72 (s, 8H, BAr$^F_4$), 7.56 (s, 4H, BAr$^F_4$), 6.79 (d, $^3J_{HH}$ = 8.1 Hz, 2H, Py), 4.65 (m, 2H, COE alkene-CH), 2.42 (virtual heptet, $^3J_{HH}$ = 7.1 Hz, 4H, C*H*(CH_3)$_2$), 1.83 – 1.38 (m, 12H, COE), 1.29 (overlapping doublets, $^3J_{HH}$ = 7.1 Hz, 24H, CH(C*H*$_3$)$_2$).

^{31}P NMR (162 MHz, CD_2Cl_2) δ 200.37 (d, $^1J_{PRh}$ = 137.6 Hz).

Elem. anal. Calcd for $C_{57}H_{57}BF_{24}NO_2P_2Rh$: C, 48.22; H, 4.05; N 0.99. Found: C, 48.15; H, 4.02; N, 1.04.

ESI-MS (CH_2Cl_2) m/z found (calculated) for $C_{25}H_{45}NO_2P_2Rh$ [M]$^+$: 556.1990 (556.1981).

Molecular structure:

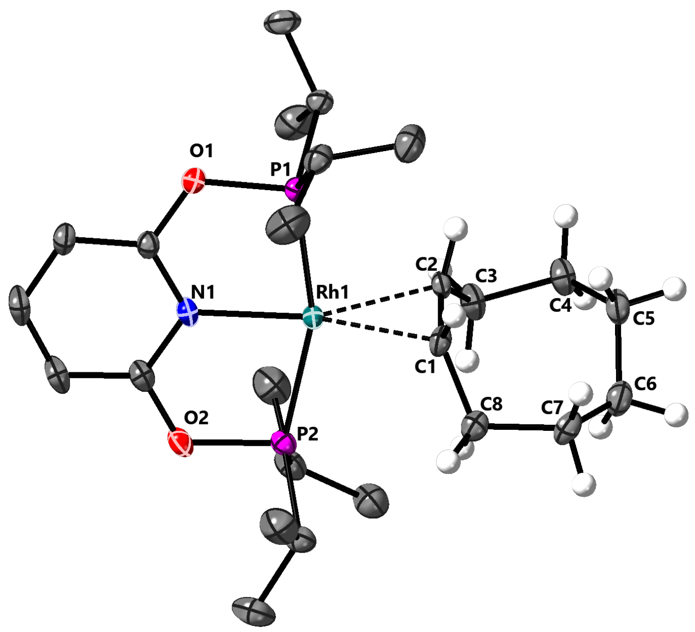

Figure S6: Molecular structure of **3** determined by single crystal X-ray diffraction. Hydrogen atoms and BAr$^F_4$ anion are omitted for clarity. Selected bond lengths [Å] and angles [°]: Rh(1)-P(1) 2.2537(6), Rh(1)-P(2) 2.3028(6), Rh(1)-N(1) 2.060(2), Rh(1)-C(1) 2.206(2), Rh(1)-C(2) 2.210(2), P(1)-O(1) 1.6750(18), P(2)-O(2) 1.692(2), C(1)-C(2) 1.360(4), C(1)-C(8) 1.510(4), C(2)-C(3) 1.501(4), C(3)-C(4) 1.542(4), C(4)-C(5) 1.536(5), C(5)-C(6) 1.529(5), C(6)-C(7) 1.523(5), C(7)-C(8) 1.539(4), P(1)-Rh(1)-P(2) 160.38(2), N(1)-Rh(1)-P(1) 80.25(6), N(1)-Rh(1)-P(2) 80.31(6), N(1)-Rh(1)-C(1) 162.04(10), N(1)-Rh(1)-C(2) 160.77(10).

Synthesis of [(^{i}Pr-PONOP)RhCl] (**6**)

To a solution of $[Rh(C_2H_4)_2Cl]_2$ (38.9 mg, 0.1 mmol) in 1,2-difluorobenzene (10 ml) was added **L1** (2.0 ml, 0.1 M soln, 0.2 mmol) and the solution stirred for 1 h. The solution was filtered by cannula, concentrated under reduced pressure to approx. 1 ml, and pentane (10 ml) added at 0 °C to precipitate a yellow solid which was collected and vacuum dried to give the product (52.5 mg, 55%).

^{1}H NMR (500 MHz, toluene-d_8) δ 6.76 (t, $^3J_{HH}$ = 8.0 Hz, 1H, Py), 6.03 (d, $^3J_{HH}$ = 8.0 Hz, 2H, Py), 2.21 (hept, $^3J_{HH}$ = 7.1 Hz, 4H, C*H*(CH$_3$)$_2$), 1.38 (m, 12H, CH(C*H*$_3$)$_2$), 1.38 (apparent quartet (dvt), 12H, *J* 7.1 Hz, CH(C*H*$_3$)$_2$).

^{31}P{^{1}H} NMR (203 MHz, toluene-d_8) δ 193.0 (d, $^1J_{PRh}$ = 154.5 Hz).

Elem. anal. Calcd for $C_{17}H_{31}NO_2P_2RhCl$: C, 42.39; H, 6.49; N 2.91. Found: C, 42.29; H, 6.46; N, 2.62.

Synthesis of [(^{i}Pr-PONOP)Rh(H_2)][BAr$^F{}_4$] (**7**)

To a high-pressure NMR tube fitted with a Young's tap (controlled atmosphere valve) were introduced [(^{i}Pr-PONOP)RhCl] (**6**) (5 mg, 10 µmol) and Na[BAr$^F{}_4$] (9 mg, 10 µmol). CD_2Cl_2 (0.4 ml) was distilled into the NMR tube and kept frozen with liquid N_2. The NMR tube was then evacuated ($p < 10^{-2}$ mbar) and repressurised with H_2 (2 bar), thawing upon anticipation of NMR analysis. The initially bright orange solution rapidly turned a darker orange-brown and a small amount of white precipitate (presumably NaCl) was observed. ^{31}P NMR spectroscopy showed 81% conversion to **7** *in situ*. Underlying impurities visible within the ^{1}H NMR spectrum obtained prevented accurate integrals being obtained for some signals associated with **7**.

^{1}H NMR (500 MHz, CD_2Cl_2, 298 K) δ 7.90 (t, $^3J_{HH}$ = 8.2 Hz, 1H, Py), 7.72 (m, BAr$^F{}_4$), 7.56 (s, BAr$^F{}_4$), 6.88 (d, $^3J_{HH}$ = 8.2 Hz, 2H, Py), 4.59 (br s, $\nu_{1/2}$ = 82 Hz, free-*H_2*), 2.42 (virtual heptet, $^3J_{HH}$ = 6.8 Hz, 4H, C*H*(CH_3)$_2$), 1.25 (m, CH(C*H_3*)$_2$), –8.38 (br s, $\nu_{1/2}$ = 150 Hz, 2H, Rh-*H_2*).

^{31}P{^{1}H} NMR (203 MHz, CD_2Cl_2, 298 K) δ 215.7 (d, $^1J_{PRh}$ 127.1 Hz).

VTNMR was conducted under H_2, with the sharpest ^{1}H NMR resonance corresponding to σ-H_2 observed at 235 K. T_1 measurements were collected on the sample at 295 K and 235 K and T_1(min) found to be 48(6) ms at 235 K, congruent with a true dihydrogen complex. Even under a pressure of H_2, the dihydrogen complex **7** was observed to slowly decompose in CD_2Cl_2 at 298 K over the course of 48 hours.

^{1}H NMR (500 MHz, CD_2Cl_2, 235 K) δ 4.33 (br s, $\nu_{1/2}$ = 43 Hz, free-*H_2*), –9.20 (br s, $\nu_{1/2}$ = 90 Hz, 2H, Rh-*H_2*).

^{31}P{^{1}H} NMR (203 MHz, CD_2Cl_2, 235 K) δ 214.7 (d, $^1J_{PRh}$ 126.1 Hz).

Upon removal of the H_2 atmosphere, the broad Rh-*H_2* signal of the dihydrogen complex **7** sharpens into a doublet with $^1J_{HRh}$ = 27.8 Hz. However, in the absence of a H_2 atmosphere, complex **7** is observed to readily decompose into a variety of [(^{i}Pr-PONOP)Rh] containing products.

Reaction of complex **7** with COE: After freeze-pump-thawing a sample of [(^{i}Pr-PONOP)Rh(H_2)][BAr$^F{}_4$] produced as above *in situ*, the NMR tube was repressurised under argon and an excess of COE (20 µl) added. The COE complex **5** was generated almost quantitatively.

Reaction of complex **7** with D_2: After freeze-pump-thawing a sample of [(^{i}Pr-PONOP)Rh(H_2)][BAr$^F{}_4$] produced as above *in situ*, the NMR tube was repressurised under D_2 (1 bar). The NMR tube was shaken vigorously and left to stand at room temperature for 10 minutes before reactivity was interrogated by NMR spectroscopy. VTNMR was conducted under D_2 and a signal corresponding to dissolved HD resolved at 255 K (δ_H 4.41 (br t, $\nu_{1/2}$ 116 Hz, $^1J_{HD}$ ~43 Hz). At 298K, the ^{2}H NMR spectrum appears silent, however, at 235 K, signals within the ^{2}H NMR spectrum corresponding to Rh-D_2/Rh-HD and dissolved D_2/HD are observed at δ_D –9.03 (*c.f.* δ_H –9.04, $\nu_{1/2}$ 152 Hz) and δ_D 4.13 ppm (*c.f.* δ_H 4.4-4.0, br overlapping signals), respectively.

1.3. Crystallography

Single-crystal X-ray diffraction data for all the compounds were collected (ω-scans) on an Oxford Diffraction/Agilent SuperNova diffractometer (Cu-Kα radiation, λ = 1.54184 Å) at the Oxford Chemical Crystallography Service from the University of Oxford equipped with nitrogen gas Oxford Cryosystems Cryostream unit.1 Diffraction data was reduced and processed using CrysAlisPro package.2 The structures were solved using SHELXT3 and refined to convergence on F2 and against all independent reflections by full-matrix least-squares using SHELXL4 (version 2018/3) in combination with the GUI OLEX25 program. All non-hydrogen atoms were refined anisotropically and hydrogen atoms were geometrically placed unless otherwise stated (see specific details for each molecular structure in the text and supplementary CIF information) and allowed to ride on their parent atoms. CF_3 groups on the $BAr^F{}_4^-$ anion were necessarily modelled as disordered over two main domains, and restrained to maintain sensible geometries. Disordered groups were restrained to maintain sensible geometries. Distances and angles were calculated using the full covariance matrix. Selected crystallographic data are summarized in Tables S1 and S2 and full details are given in the supplementary deposited CIF files (CCDC). These data can be obtained free of charge from the Cambridge Crystallographic Data Centre via http://optimized.ccdc.cam.ac.uk/data_request/cif.

Table S1. Selected crystallographic and refinement data

	**1**	**2**	**3**
CCDC number	2050110	2050111	2050112
Formula	$C_{57}H_{55}BF_{24}NO_2P_2Rh$	$C_{56}H_{51}BF_{24}NO_2P_2Rh$	$C_{51}H_{47}BF_{24}NO_2P_2Rh$
M	1417.68	1401.63	1337.55
Crystal System	Triclinic	Triclinic	Triclinic
Space group	*P*-1	*P*-1	*P*-1
T [K]	150.01(10)	150.01(12)	150.01(10)
a [Å]	12.9544(3)	12.80500(10)	12.1476(3)
b [Å]	13.0636(3)	25.2728(3)	12.1596(3)
c [Å]	19.6051(4)	28.8265(3)	20.0616(4)
α [°]	73.940(2)	101.7660(10)	78.274(2)
β [°]	73.960(2)	101.7990(10)	78.335(2)
γ [°]	83.555(2)	90.4000(10)	89.884(2)
V [$Å^3$]	3061.88(13)	8928.26(16)	2839.17(12)
Z	2	6	2
Density [gcm^{-3}]	1.538	1.564	1.565
μ [mm^{-1}]	3.788	3.891	4.047
θ range [°]	2.427 to 74.910	3.531 to 76.349	2.299 to 77.295
Reflns collected	44529	167726	52058
R_{int}	0.0585	0.0390	0.0758
Compl. 67.684 °.	100%	100%	100%
Data/restr/param	12354 / 813 / 969	36810 / 3634 / 3381	11910 / 405 / 831
R_1 [I > 2σ(I)]	0.0581	0.0551	0.0565
wR_2 [all data]	0.1483	0.1525	0.1608
GoF	1.037	1.026	1.032
Larg. pk / hole [$eÅ^{-3}$]	0.973 and -0.852	2.391 and -1.365	1.862 and -1.144

Table S2. Selected crystallographic and refinement data

	**4**	**5**
CCDC number	2050113	2050114
Formula	$C_{49}H_{43}AgBF_{24}NO_2P_2$	$C_{57}H_{57}BF_{24}NO_2P_2Rh$
M	1314.46	1419.69
Crystal System	Triclinic	Triclinic
Space group	*P*-1	*P*-1
T [K]	150.0(3)	150.00(16)
a [Å]	13.1321(2)	12.8615(4)
b [Å]	14.4062(3)	13.1578(2)
c [Å]	14.7988(3)	19.8679(5)
α [°]	91.337(2)	73.554(2)
β [°]	98.964(2)	73.226(3)
γ [°]	95.343(2)	83.261(2)
V [$Å^3$]	2751.44(9)	3085.13(14)
Z	2	2
Density [gcm^{-3}]	1.587	1.528
μ [mm^{-1}]	4.592	3.760
θ range [°]	3.424 to 76.332	3.592 to 76.193
Reflns collected	56118	33040
R_{int}	0.0274	0.0334
Compl. 67.684 °.	100%	100%
Data/restr/param	11394 / 558 / 841	12770 / 696 / 941
R_1 [I > 2σ(I)]	0.0382	0.0398
wR_2 [all data]	0.0967	0.1041
GoF	1.030	1.037
Larg. pk / hole [$eÅ^{-3}$]	1.096 and -1.189	0.808 and -0.508

1.4. Angles between planes – alkene complexes

β is the angle between the plane containing the alkene C and Rh and the plane containing the P atoms and Rh.

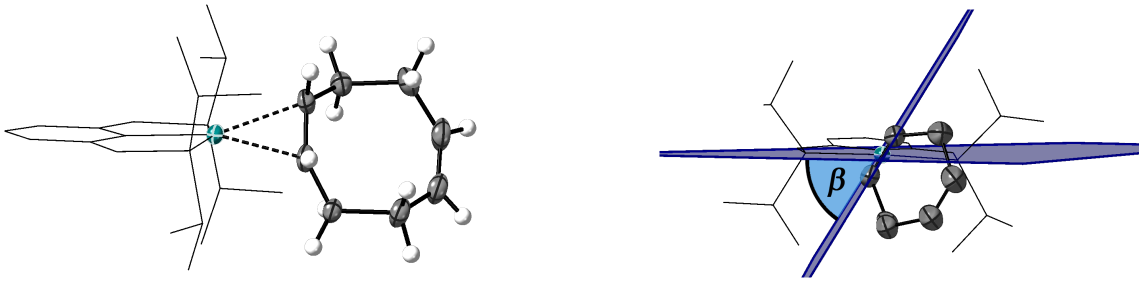

Figure S7: Complex **1**, *β* = 58.641°

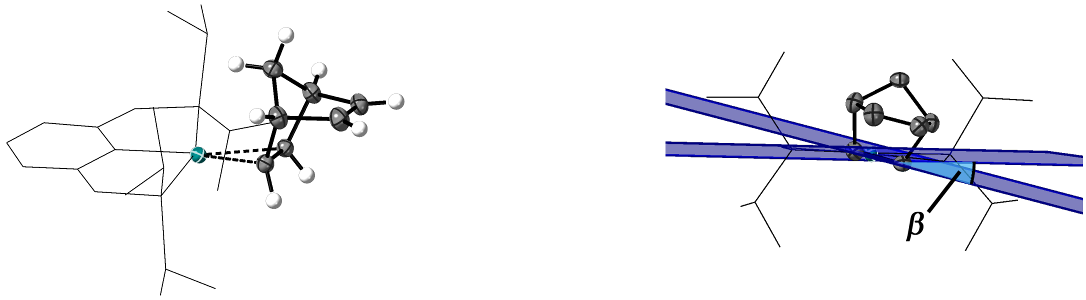

Figure S8: Complex **2**, *β* = 13.318°

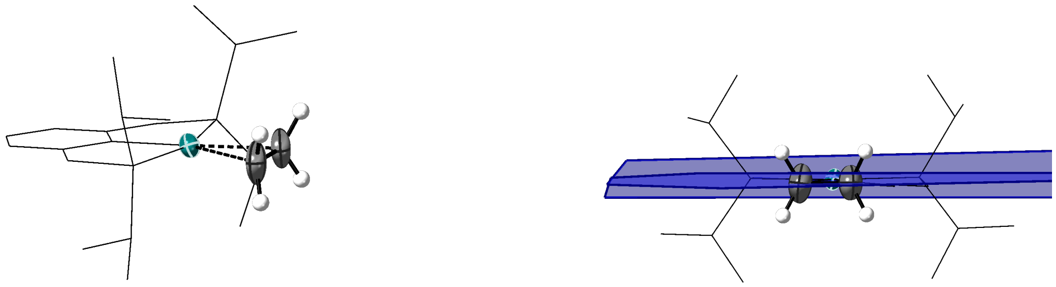

Figure S9: Complex **3**, *β* = 4.642°

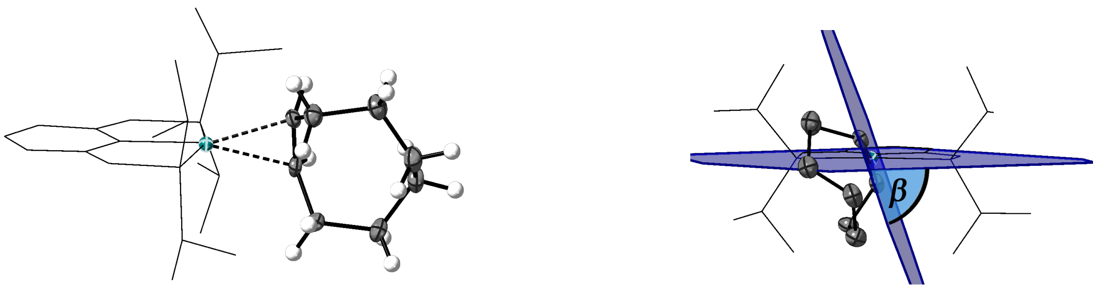

Figure S10: Complex **5**, *β* = 69.774°

1.5. NMR Spectra for iPr-PONOP (L1)

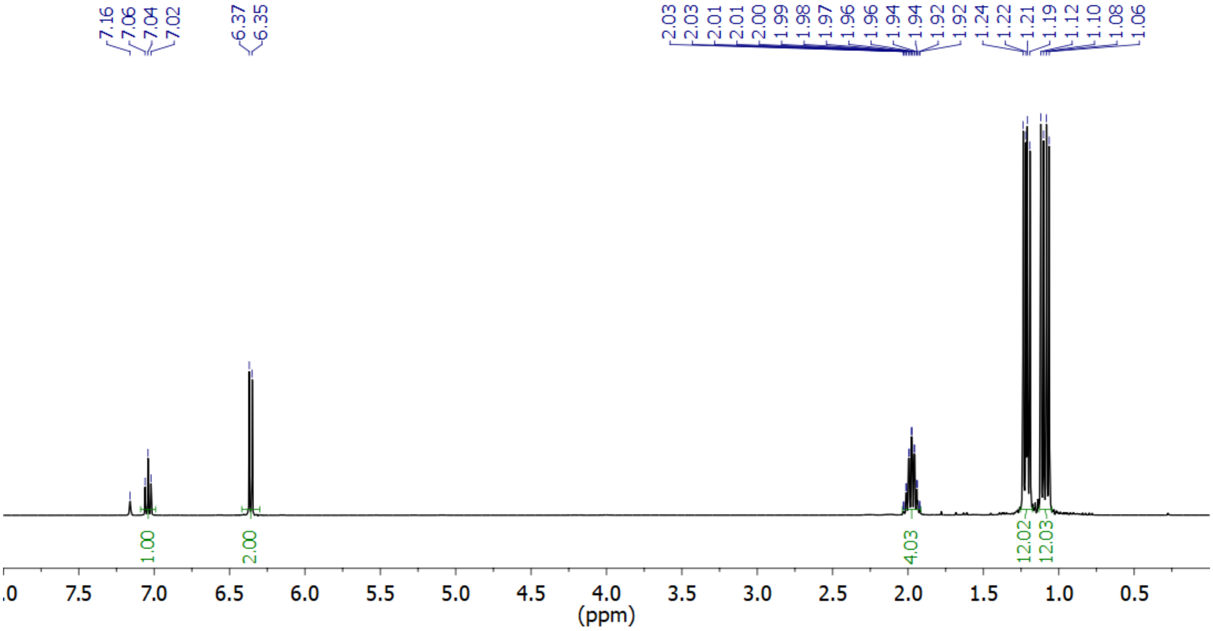

Figure S11: ^{1}H NMR (400 MHz, C_6D_6, 298 K)

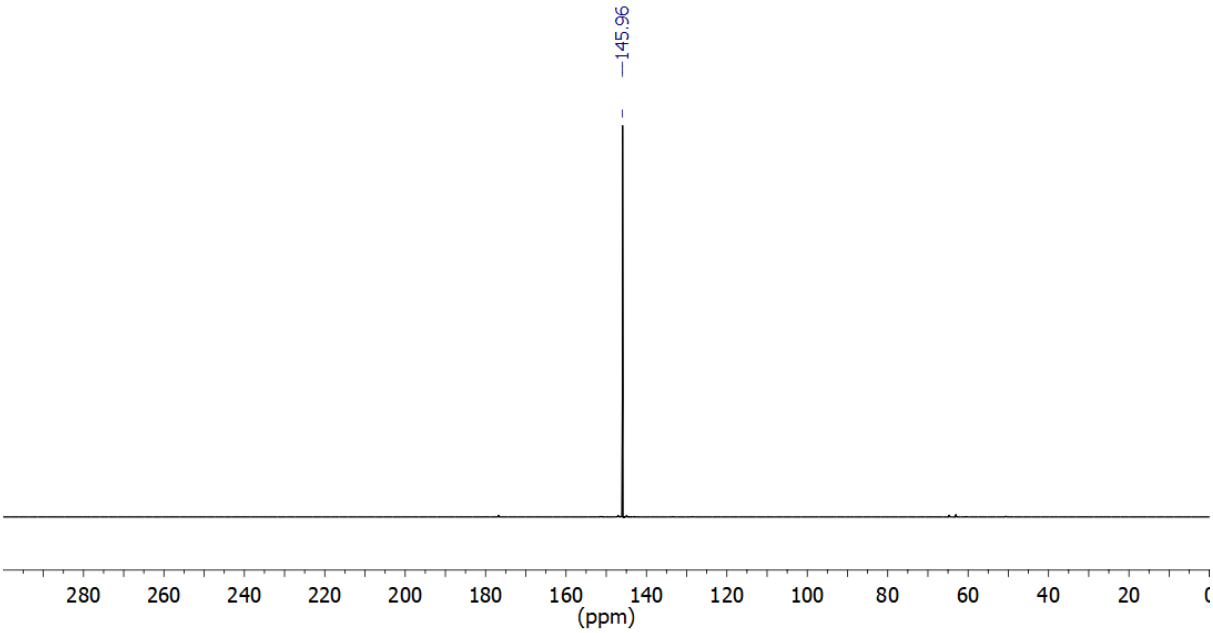

Figure S12: ^{31}P NMR (162 MHz, C_6D_6, 298 K)

1.6. NMR Spectra for 1

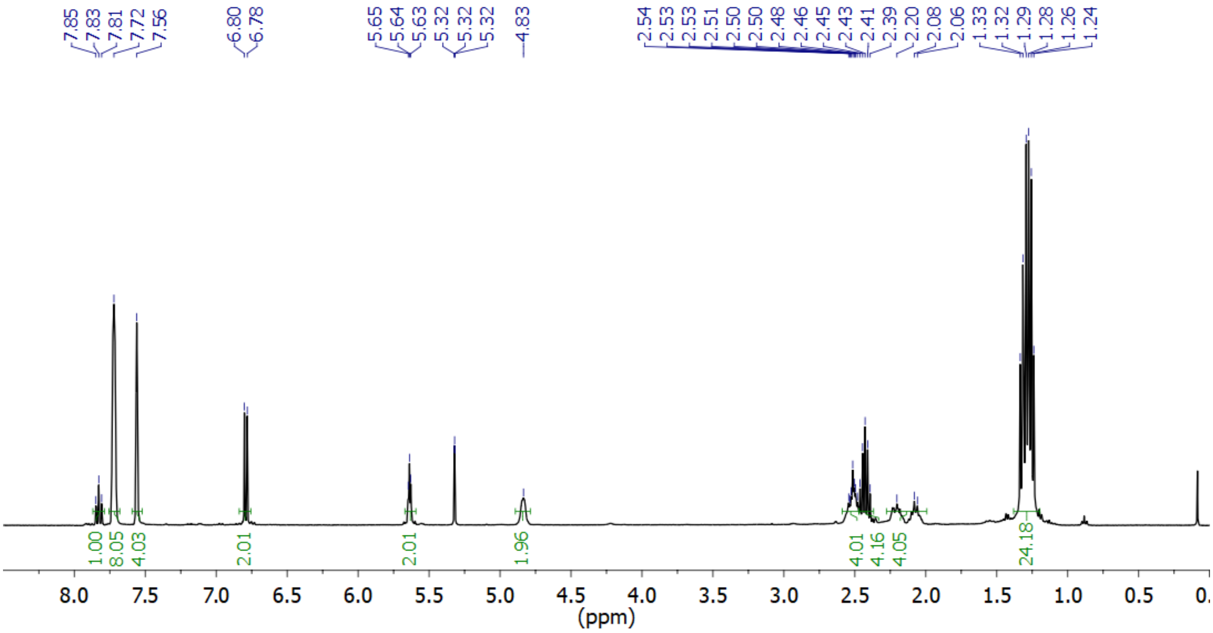

Figure S13: ^{1}H NMR (400 MHz, CD_2Cl_2, 298 K)

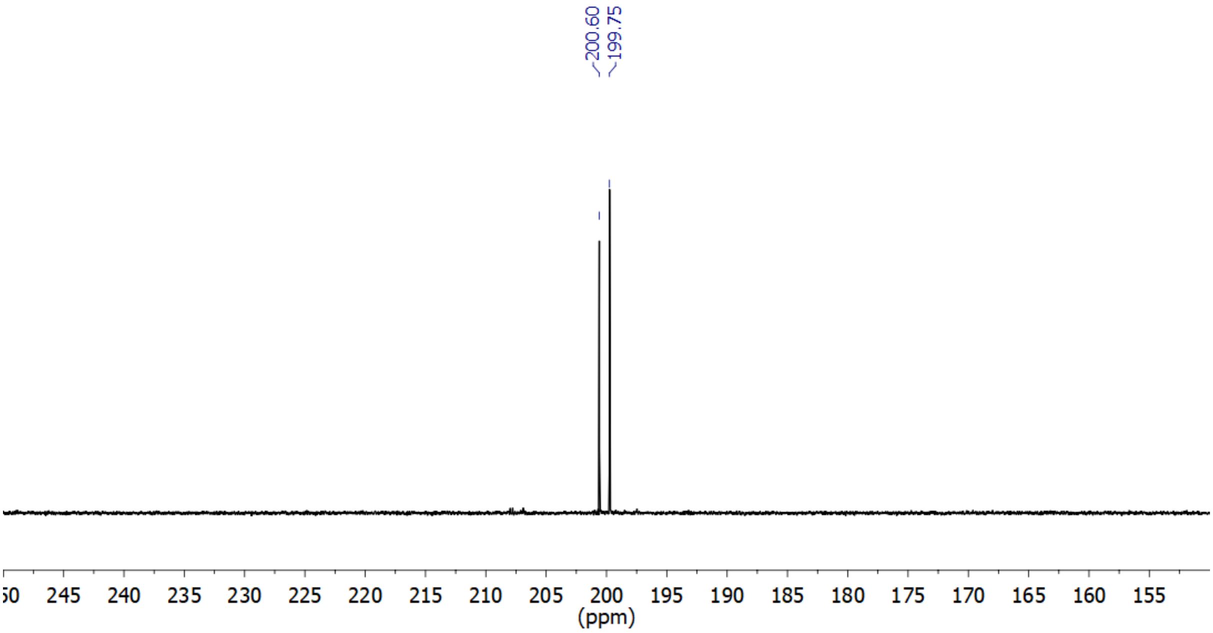

Figure S14: ^{31}P NMR (162 MHz, CD_2Cl_2, 298 K)

1.7. NMR Spectra for 2

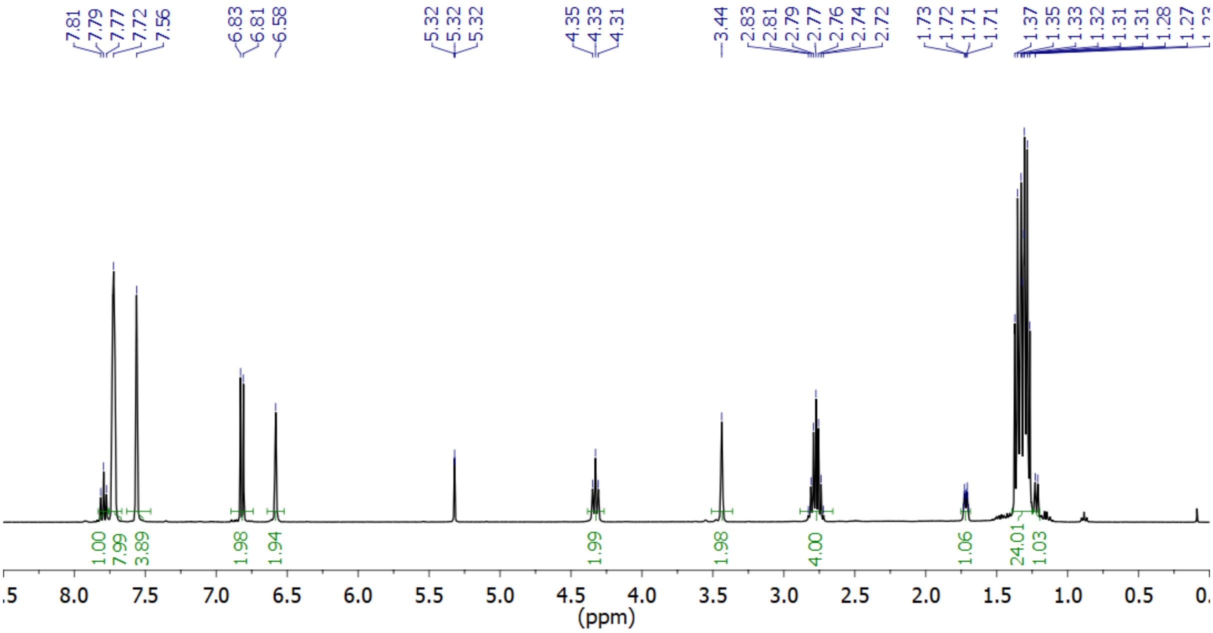

Figure S15: ^{1}H NMR (400 MHz, CD_2Cl_2, 298 K)

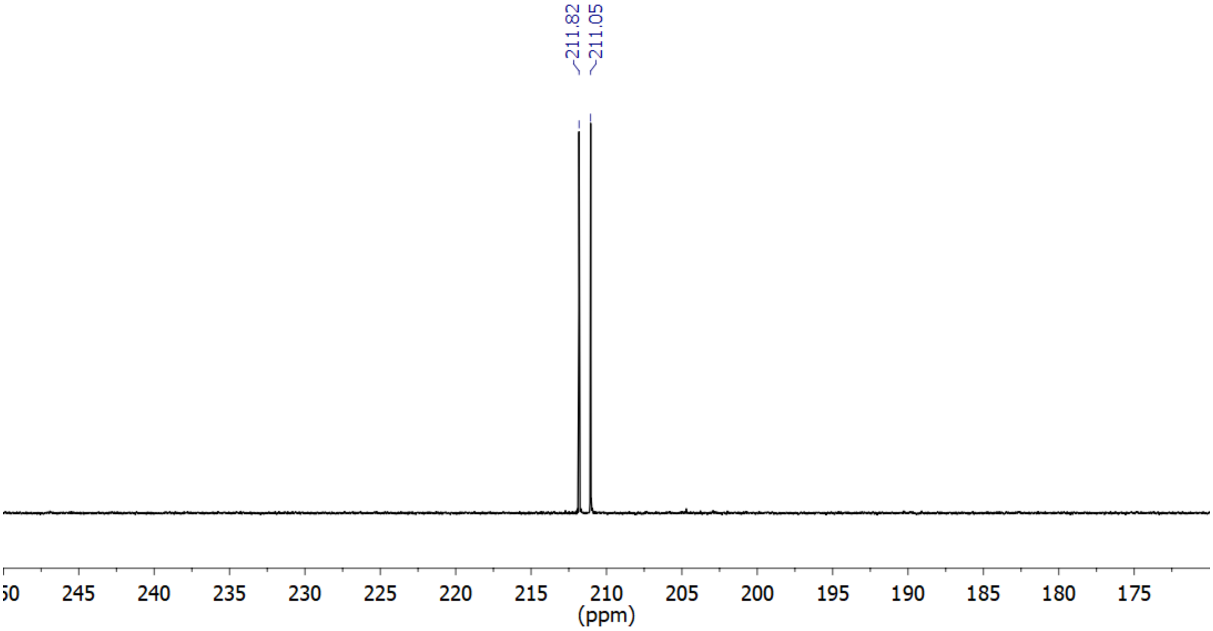

Figure S16: ^{31}P NMR (162 MHz, CD_2Cl_2, 298 K)

1.8. NMR Spectra for 3

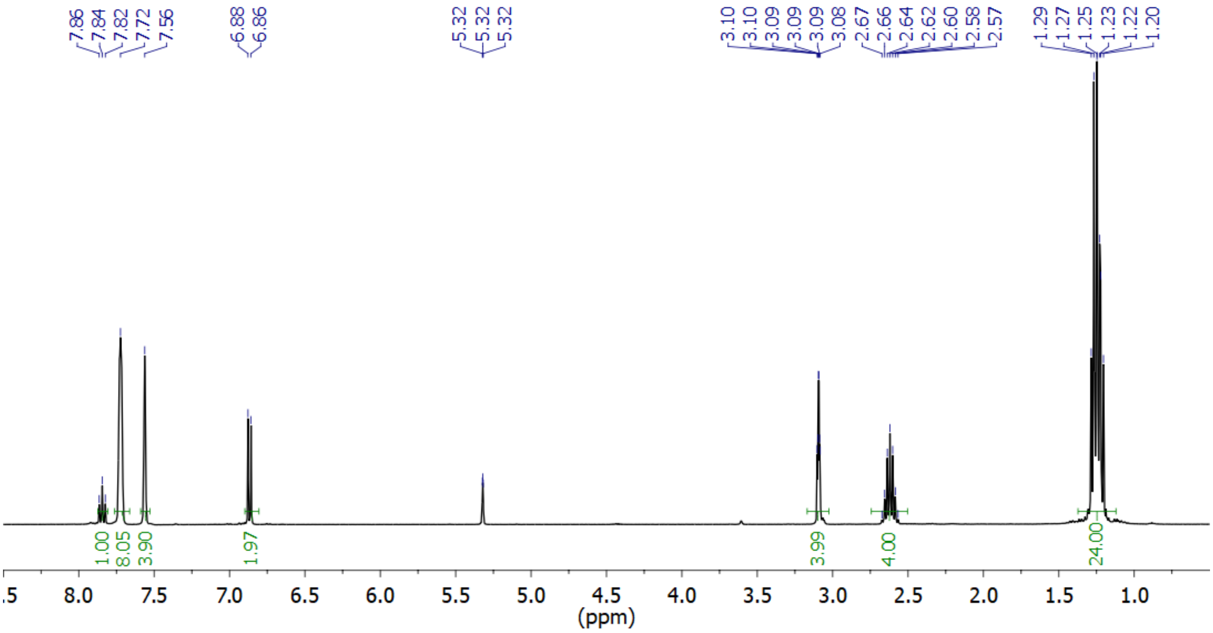

Figure S17: ^{1}H NMR (400 MHz, CD_2Cl_2, 298 K)

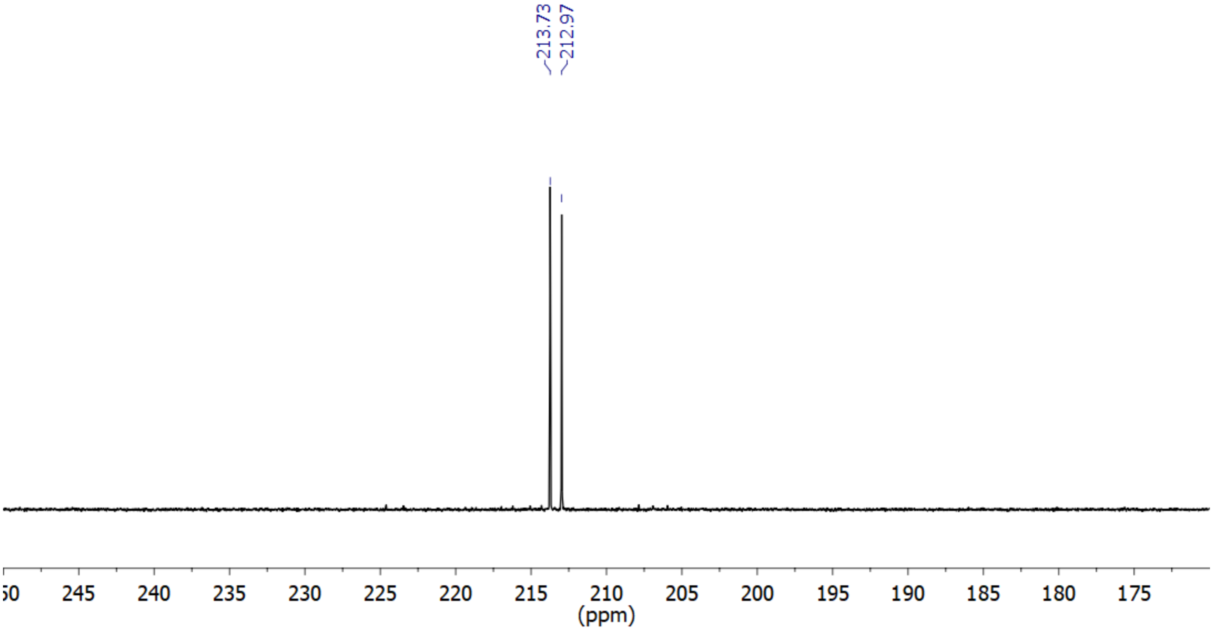

Figure S18: ^{31}P NMR (162 MHz, CD_2Cl_2, 298 K)

1.9. NMR Spectra for 4

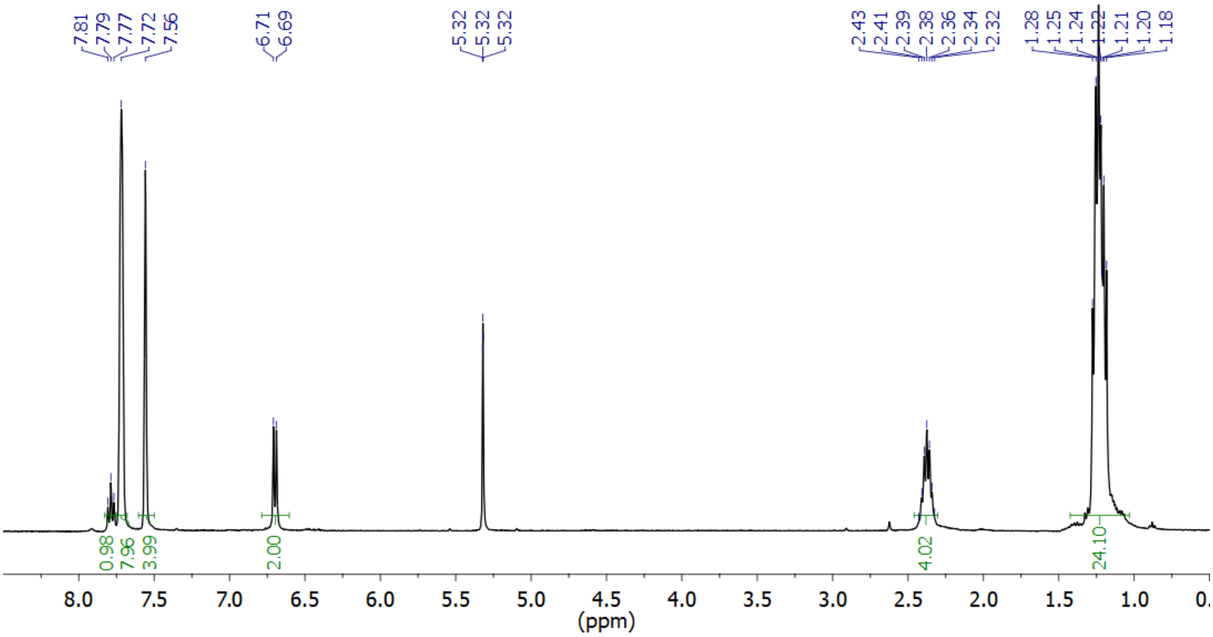

Figure S19: ^{1}H NMR (400 MHz, CD_2Cl_2, 298 K)

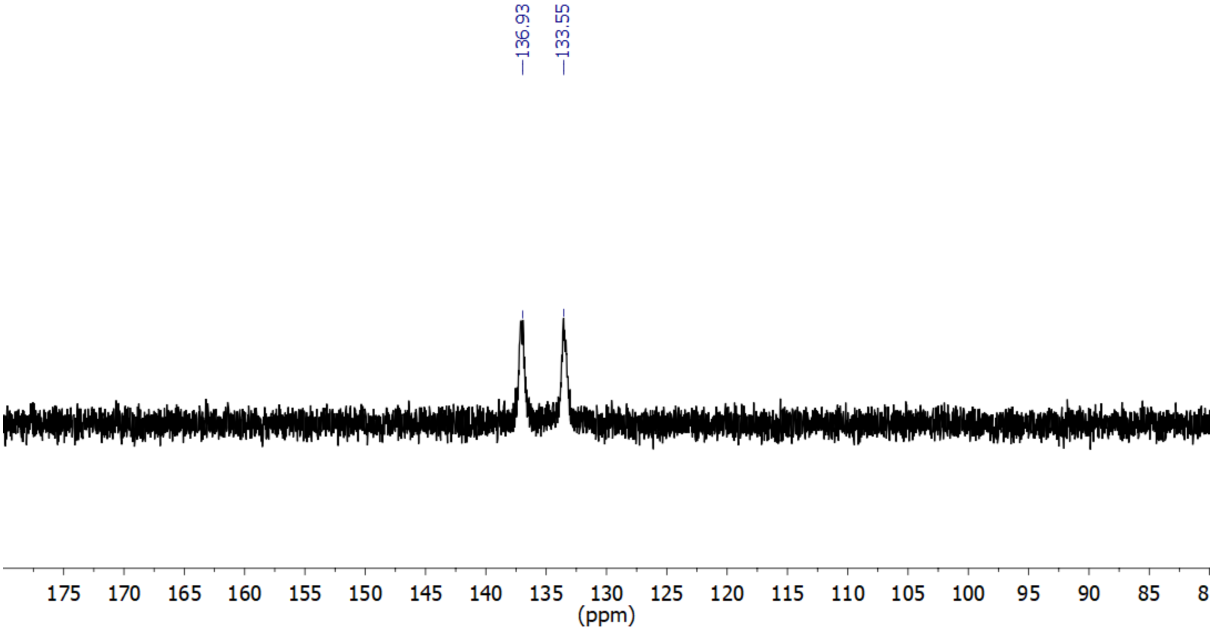

Figure S20: ^{31}P NMR (162 MHz, CD_2Cl_2, 298 K)

1.10. NMR Spectra for 5

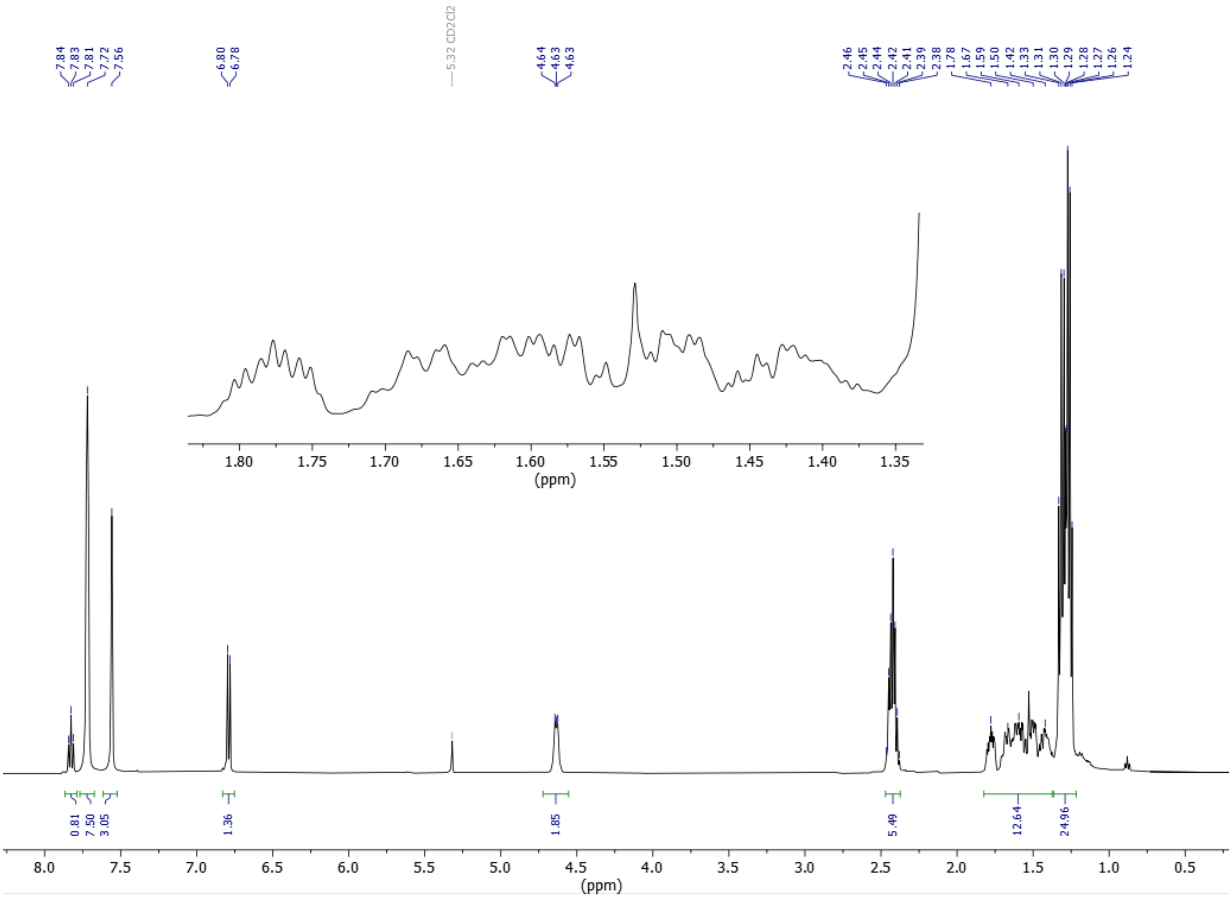

Figure S21: ^{1}H NMR (400 MHz, CD_2Cl_2, 298 K)

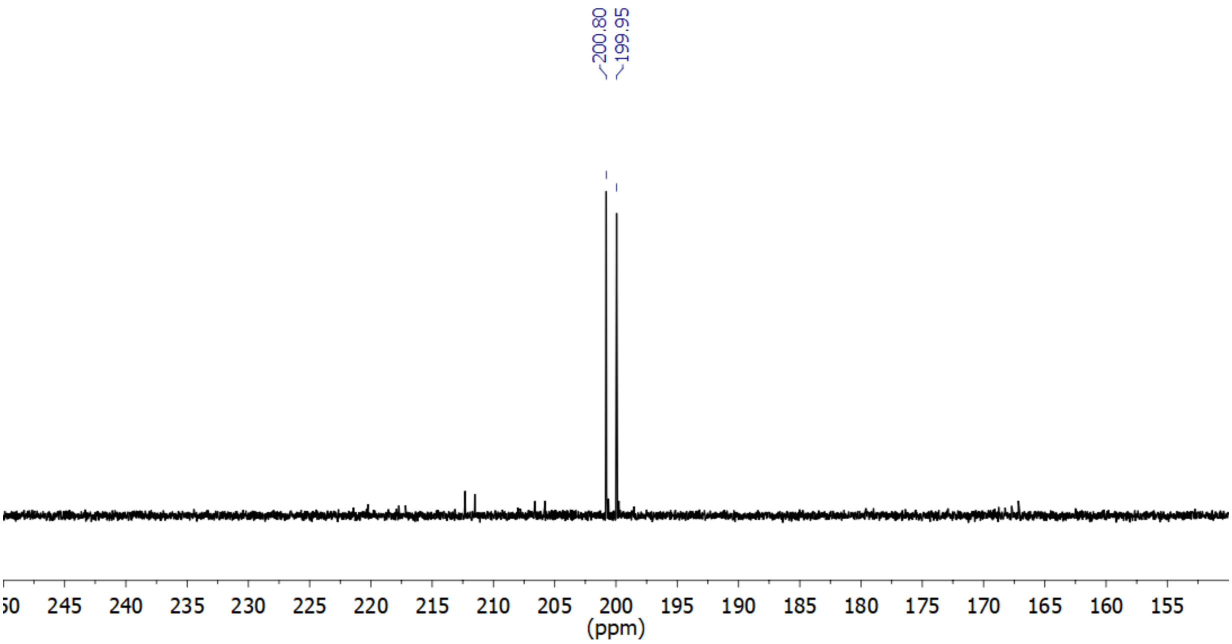

Figure S22: ^{31}P NMR (162 MHz, CD_2Cl_2, 298 K)

1.11. NMR Spectra for 6

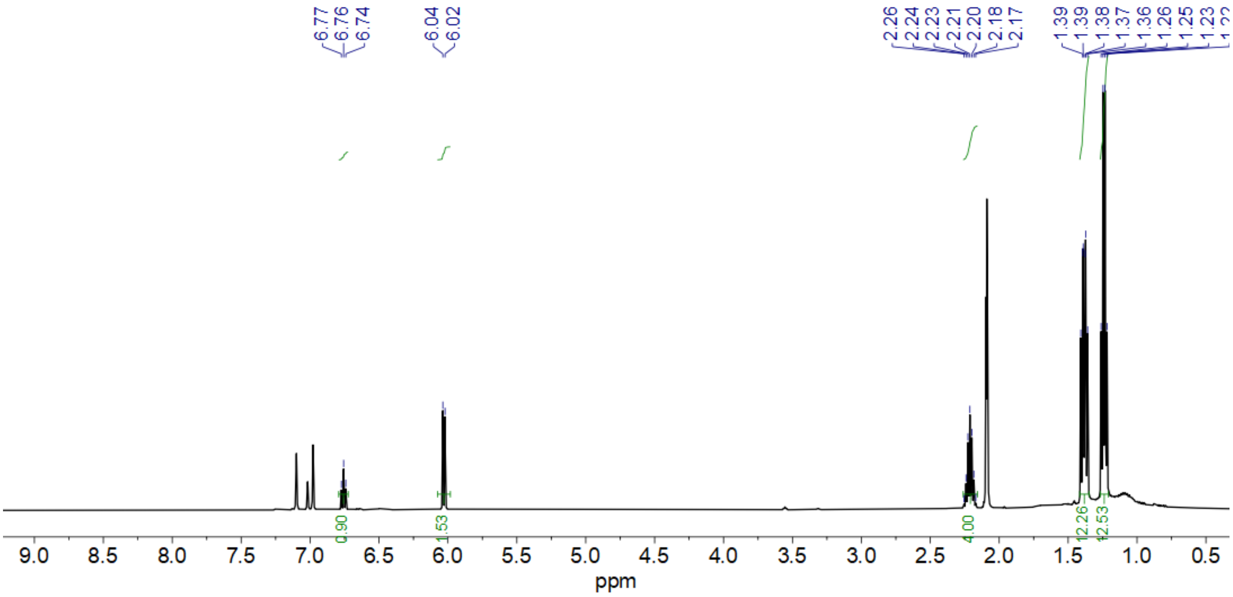

Figure S23: ^{1}H NMR (500 MHz, d_8-toluene, 298 K)

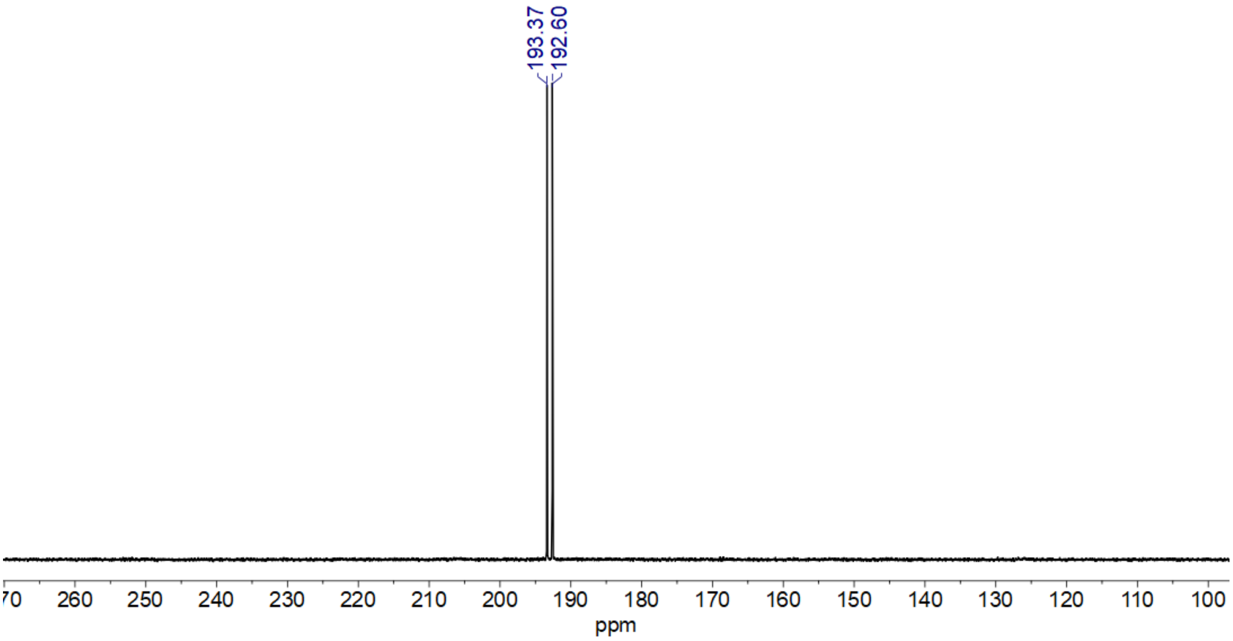

Figure S24: $^{31}P\{^{1}H\}$ NMR (203 MHz, d_8-toluene, 298 K)

1.12. NMR Spectra for 7 under an H_2 atmosphere

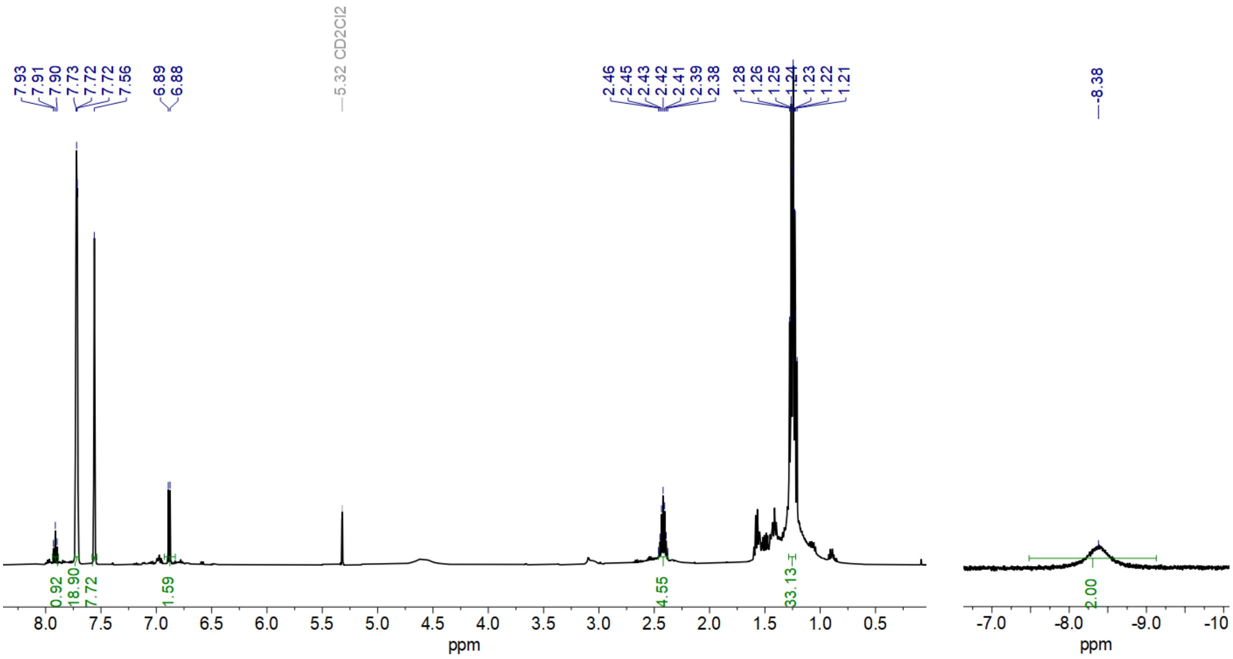

Figure S25: ^{1}H NMR (400 MHz, CD_2Cl_2, 298 K)

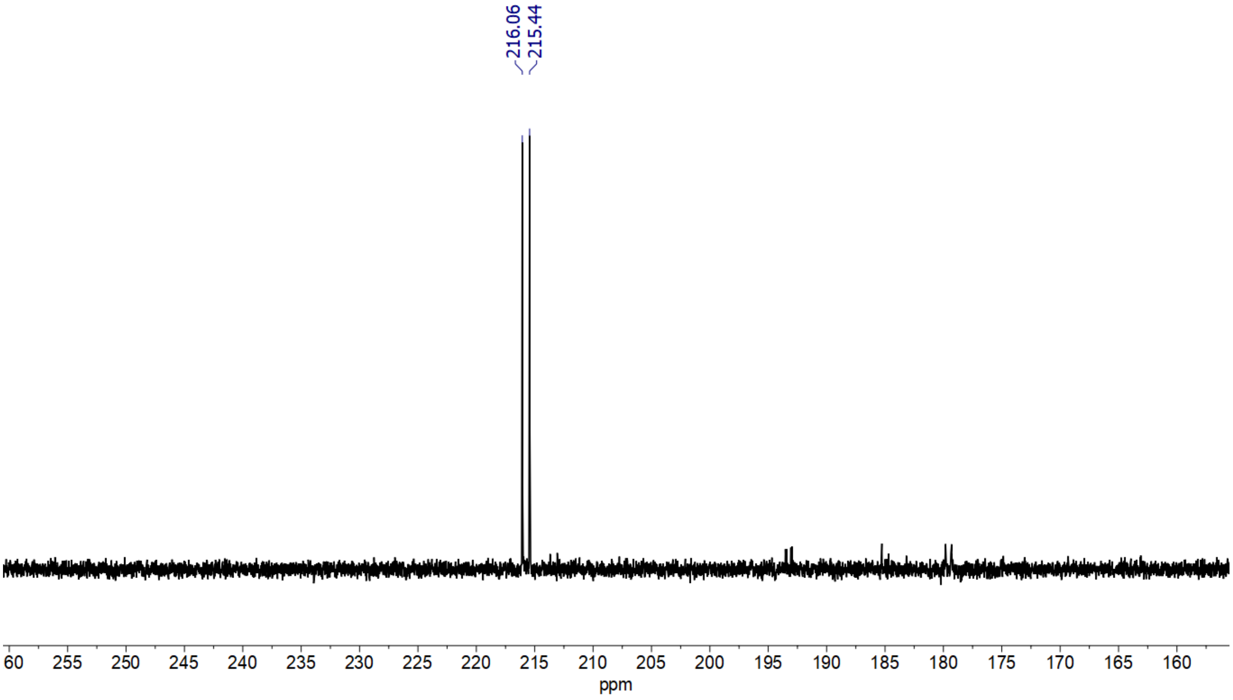

Figure S26: $^{31}P\{^{1}H\}$ NMR (162 MHz, CD_2Cl_2, 298 K)

1.13. NMR Spectra for 7 in the absence of an H_2 atmosphere

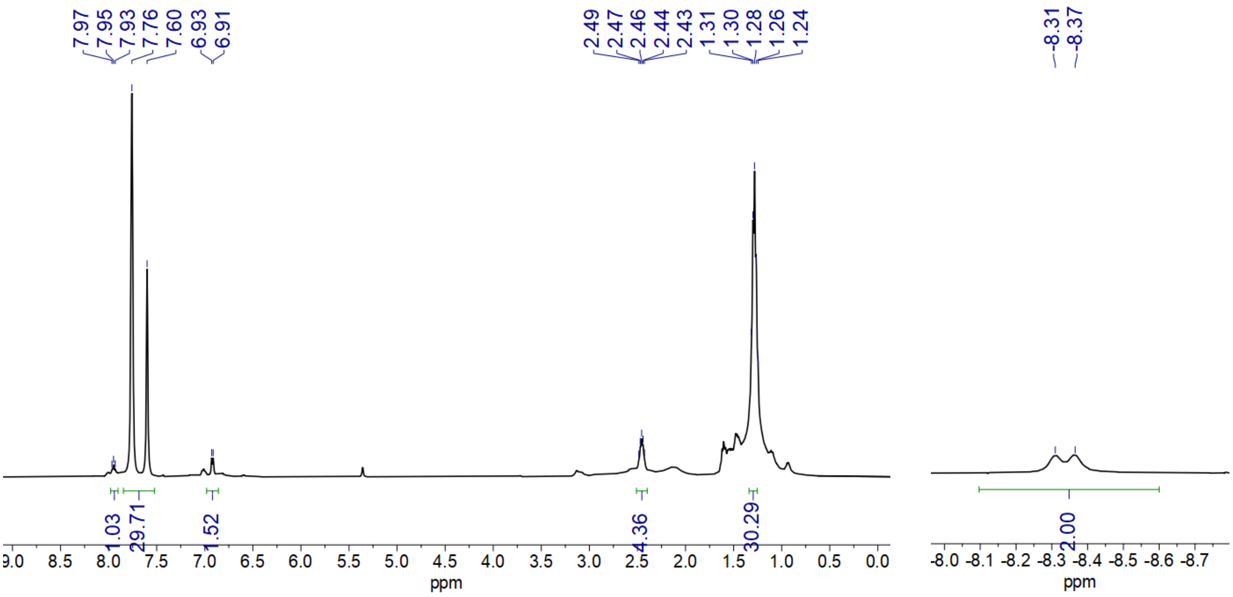

Figure S27: ^{1}H NMR (500 MHz, CD_2Cl_2, 298 K)

Removal of the H_2 atmosphere present for the synthesis of complex **7** *in situ* leads to decomposition of the sample into multiple [(^{i}Pr-PONOP)Rh] containing species:

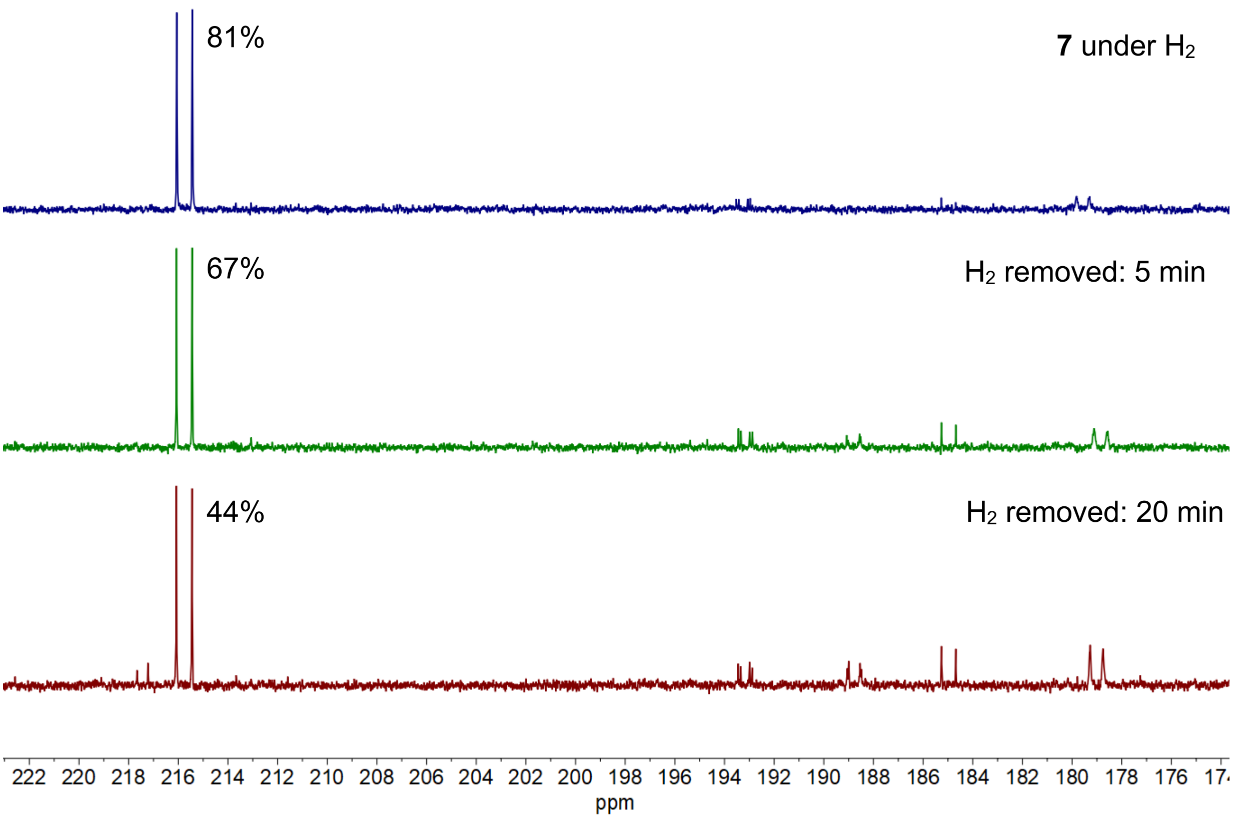

Figure S28: $^{31}P\{^{1}H\}$ NMR (203 MHz, CD_2Cl_2, 298 K)

1.14. Variable Temperature ^{31}P NMR Complex 4

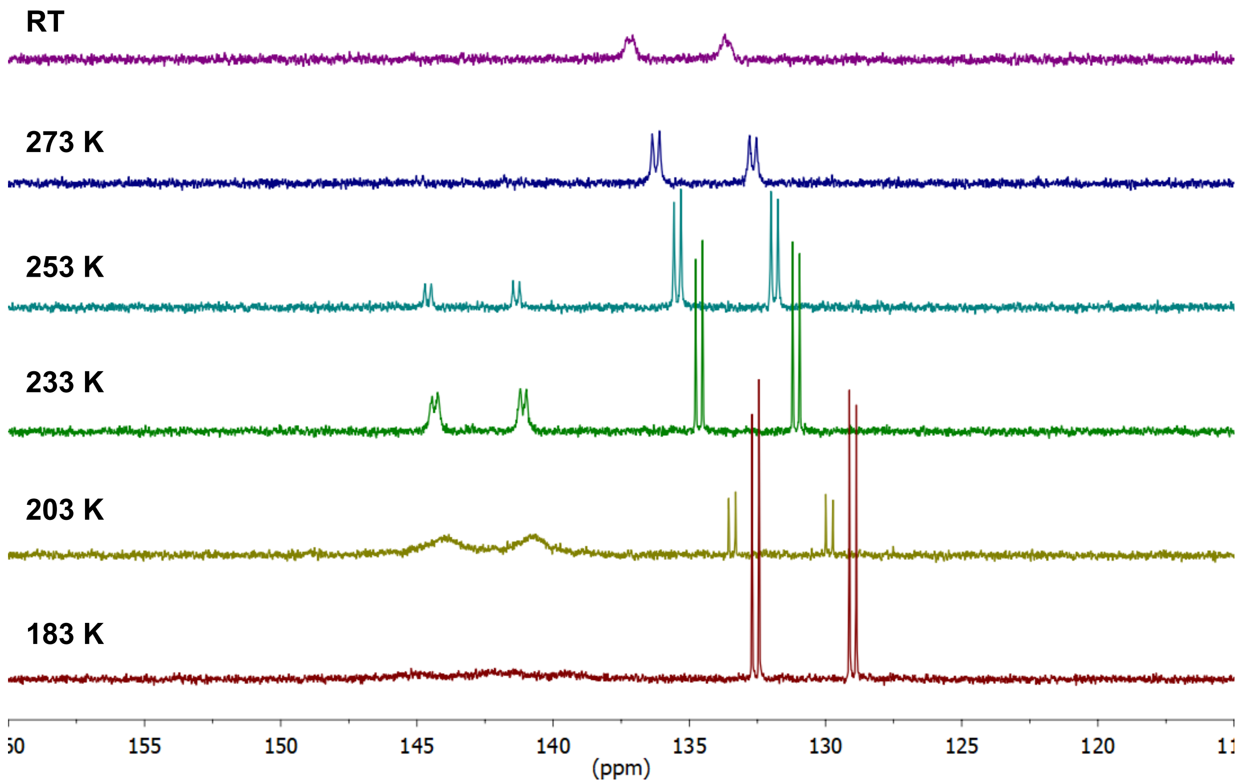

Figure S29: ^{1}H VTNMR (400 MHz, CD_2Cl_2)

1.15. Variable Temperature ^{1}H NMR Complex 7

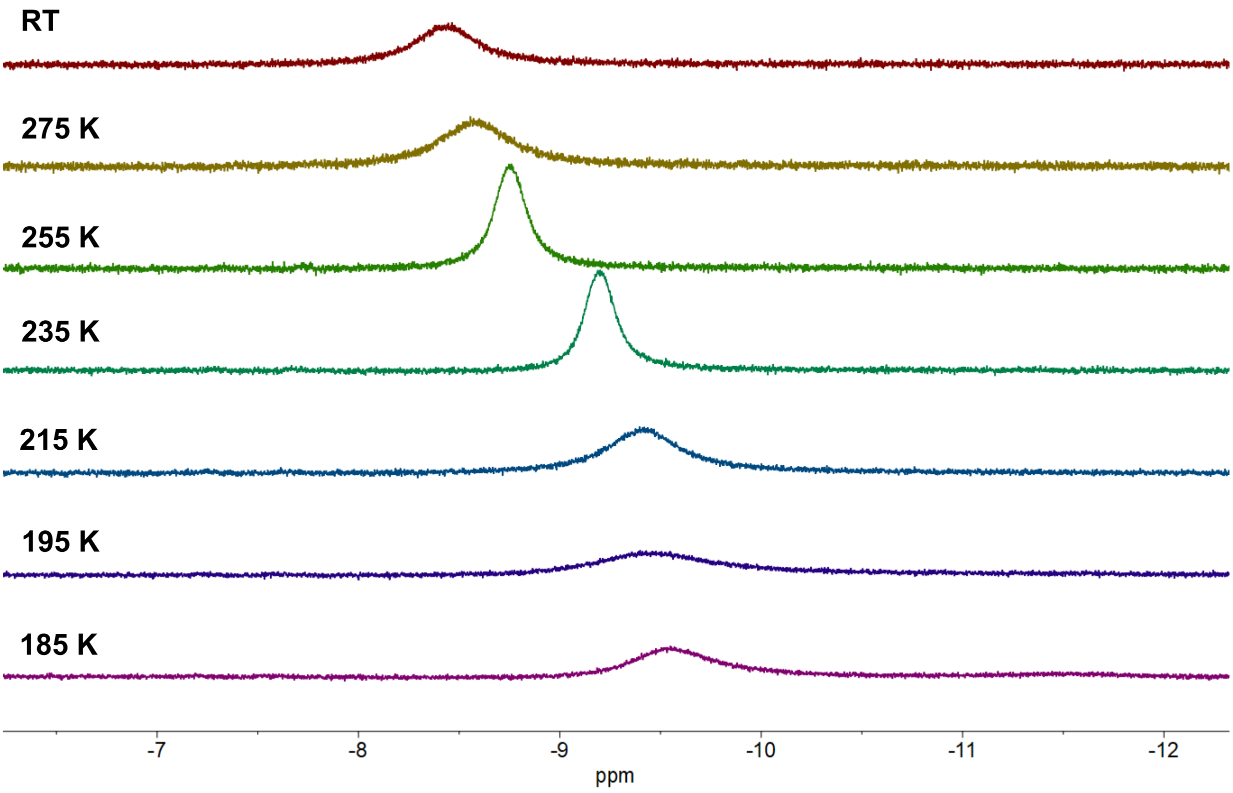

Figure S30: ^{1}H VTNMR (500 MHz, CD_2Cl_2)

1.16. Variable Temperature ^{1}H NMR Spectra for the reaction of Complex 7 with D_2

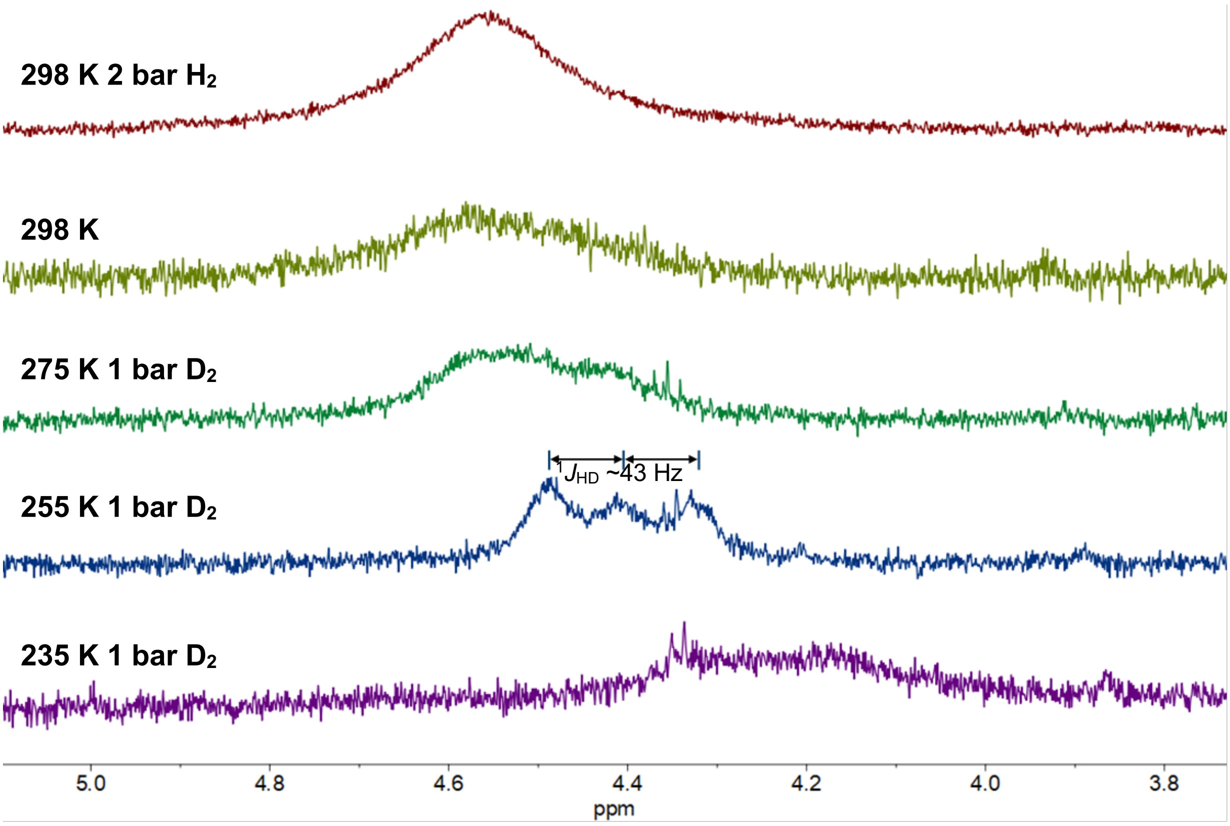

Figure S31: ^{1}H VTNMR (500 MHz, CD_2Cl_2)

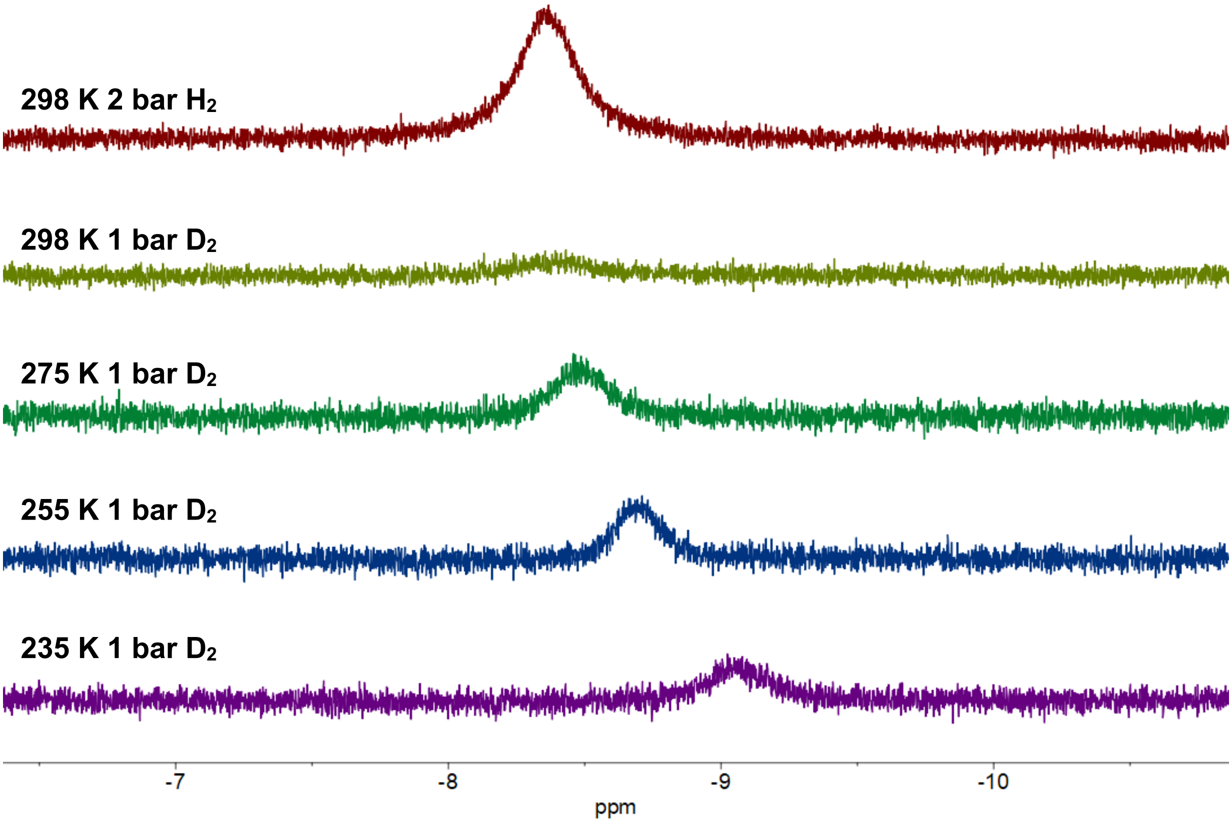

Figure S32: ^{1}H VTNMR (500 MHz, CD_2Cl_2)

1.17. Variable Temperature ^2H NMR Spectra for the reaction of Complex 7 with D_2

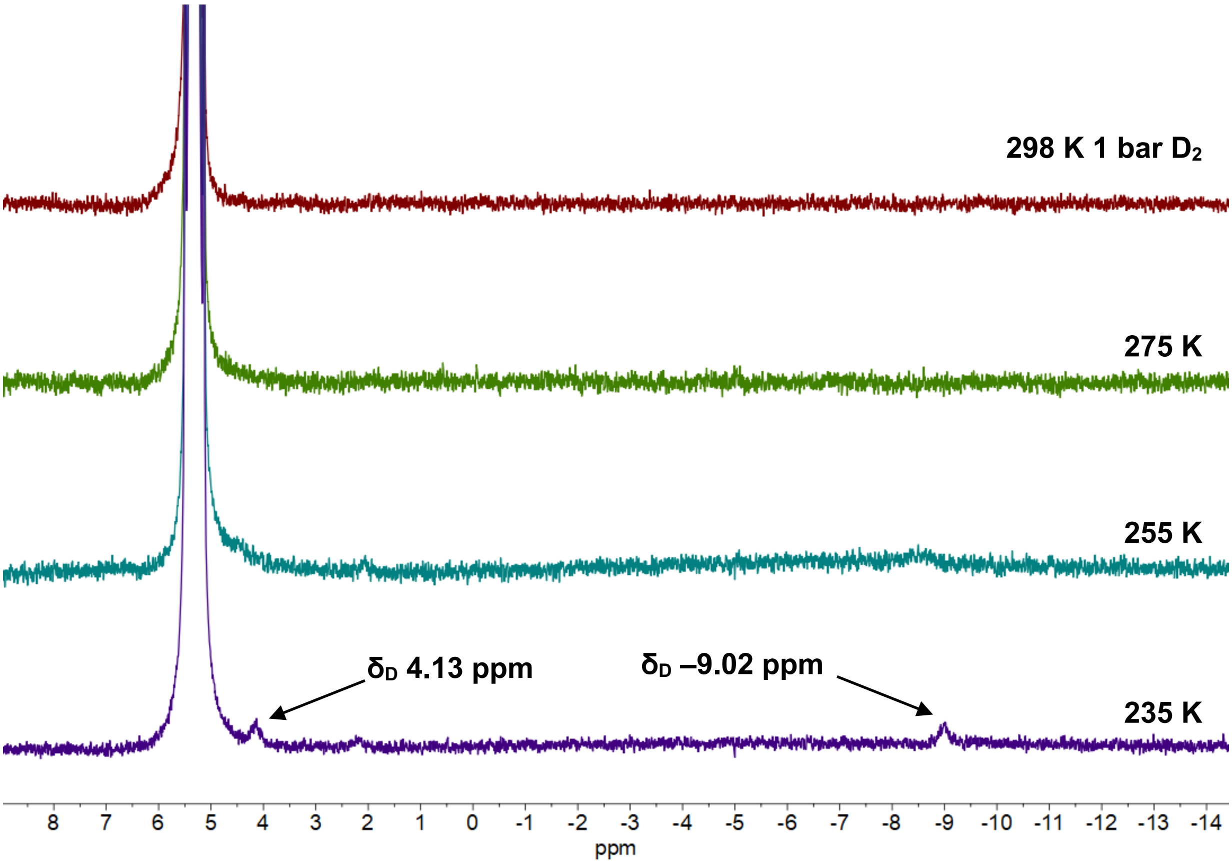

Figure S33: ^2H VTNMR (76.79 MHz, CD_2Cl_2)

1.18. Hydrogenation of Complex 1

In a high pressure NMR tube a solution of **1** (10 mg) in CD_2Cl_2 (0.4 ml) was placed under an atmosphere of H_2 (1 atm, freeze-pump-thaw cycles) and the solution stirred (NMR tube spinner). NMR spectra were recorded after 10 min and after 2 h showing conversion to complex **5**.

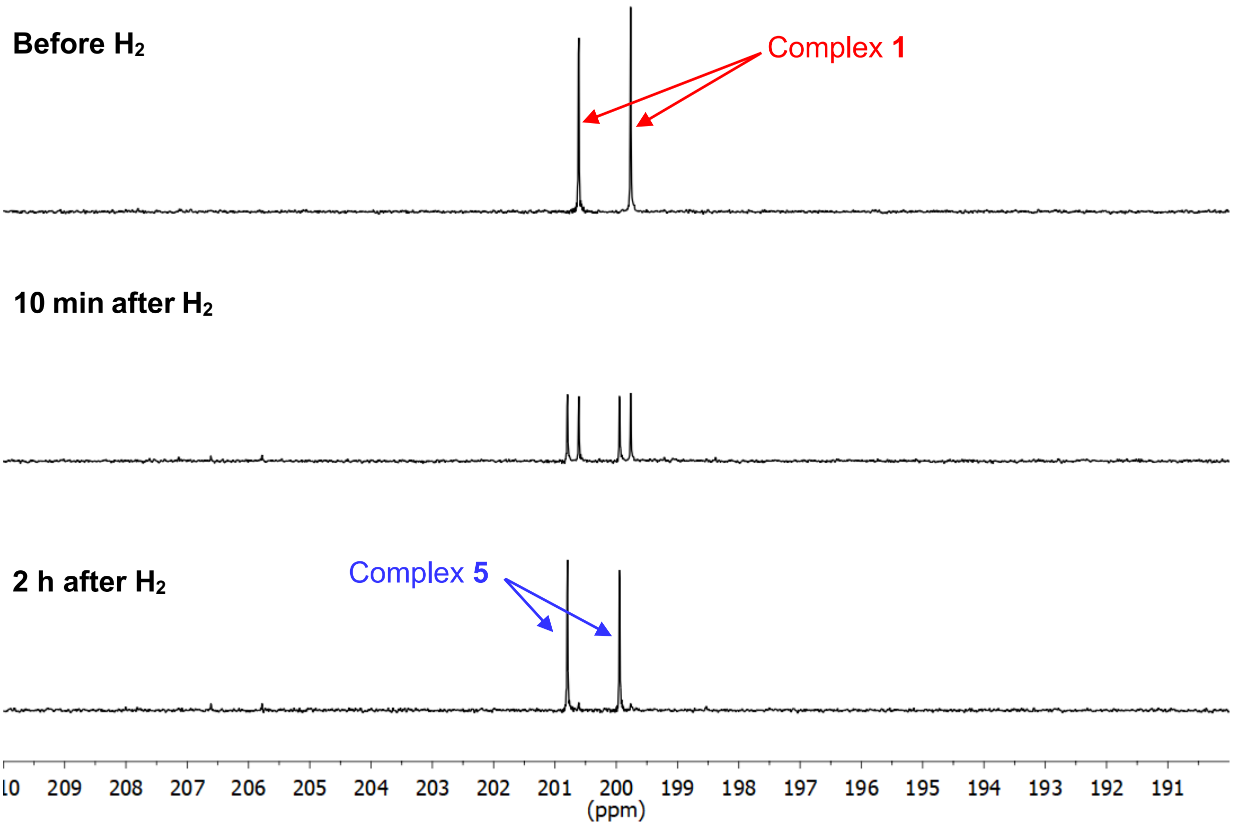

Figure S34: $^{31}P\{^1H\}$ NMR (162 MHz, CD_2Cl_2, 298 K)

1.19. Catalytic Hydrogenation of COD with Complex 1 as Precatalyst

In a high-pressure NMR tube was added COD (24.5 µl, 0.2 mmol) and **1** (14.3 mg, 0.01 mmol, 5 mol%) in CD_2Cl_2 (0.4 ml). The solution was placed under an atmosphere of H_2 (1 atm., freeze-pump-thaw cycles) and the solution mixed (NMR tube spinner). NMR spectra were recorded after different time intervals.

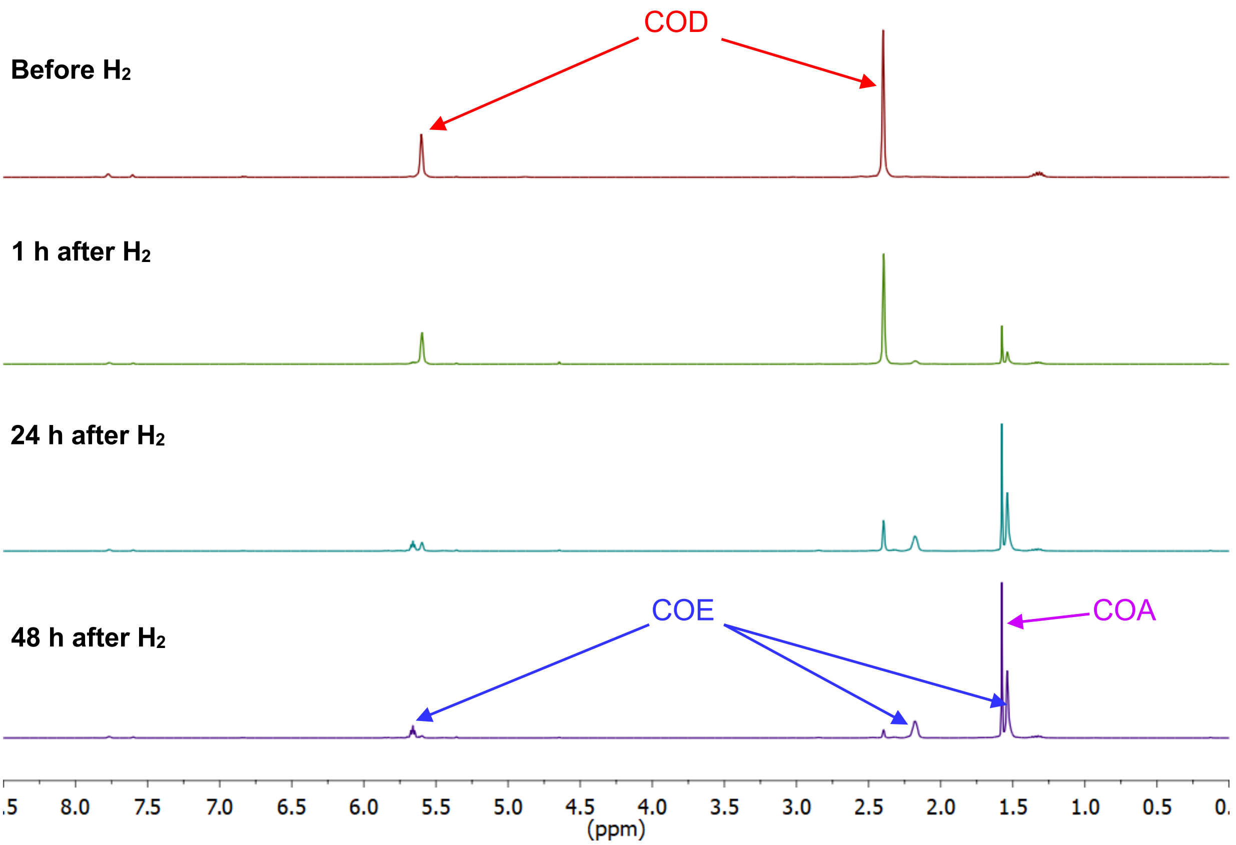

Figure S35: ^{1}H NMR (400 MHz, CD_2Cl_2, 298 K)

1.20. Catalytic Hydrogenation of COE with Complex 3 as Precatalyst

In a high-pressure NMR tube **1** (14.3 mg, 0.01 mmol) was dissolved in CD_2Cl_2 (0.4 ml) and the solution placed under an atmosphere of H_2 (1 atm., freeze-pump-thaw cycles) and stirred (NMR tube spinner). Upon complete conversion to **3** as confirmed by NMR experiments (after 1 h reaction), COE (25 µl, 0.19 mmol) was added and the solution placed under an atmosphere of H_2 (1 atm., freeze-pump-thaw cycles) and stirred (NMR tube spinner). NMR spectra were recorded after different time intervals.

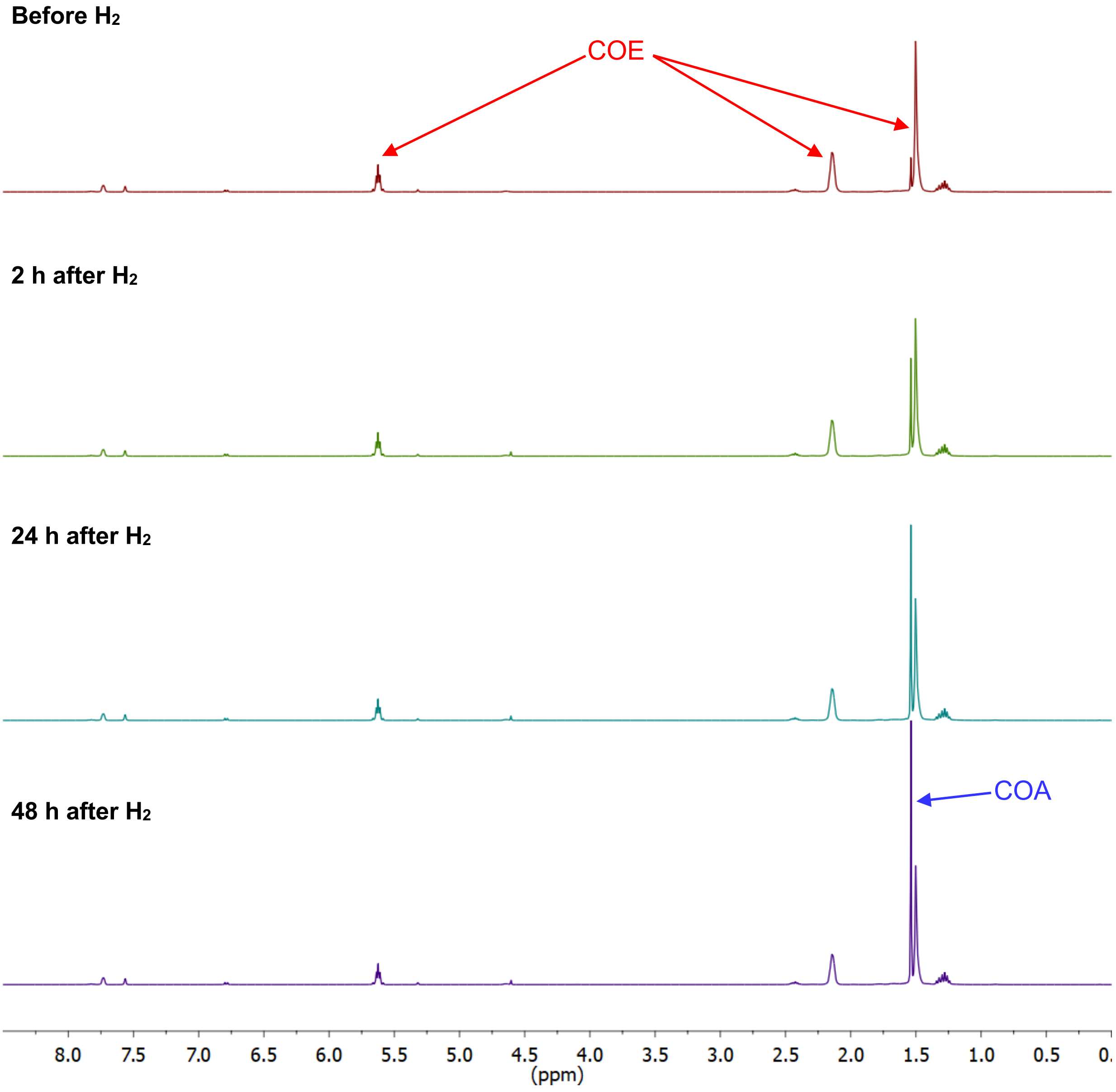

Figure S36: ^{1}H NMR (400 MHz, CD_2Cl_2, 298 K)

1.21. Catalytic Hydrogenation of C_2H_4 with Complex 5 as Precatalyst

Complex **3** (1 mg, 0.7 µmol) was introduced to a high-pressure NMR tube and dissolved in CD_2Cl_2 (0.4 ml). The solution was then frozen with liquid N_2, exposed to dynamic vacuum ($p < 10^{-2}$ bar), closed to vacuum, and allowed to warm to room temperature before the tube was pressurised with ethene (1 bar) and closed. The solution was then frozen again with liquid N_2, exposed to dynamic vacuum ($p < 10^{-2}$ bar), closed to vacuum, and repressurised with H_2 (1 bar). After thawing, ^{1}H NMR studies were commenced immediately to determine the conversion of ethene to ethane in CD_2Cl_2 solution (**Figure S37**). Interrogation of the solution by ^{1}H NMR at this initial stage revealed *c.* 183 equivalents of ethene in solution *versus* the catalyst (*i.e.* 0.55 mol%).

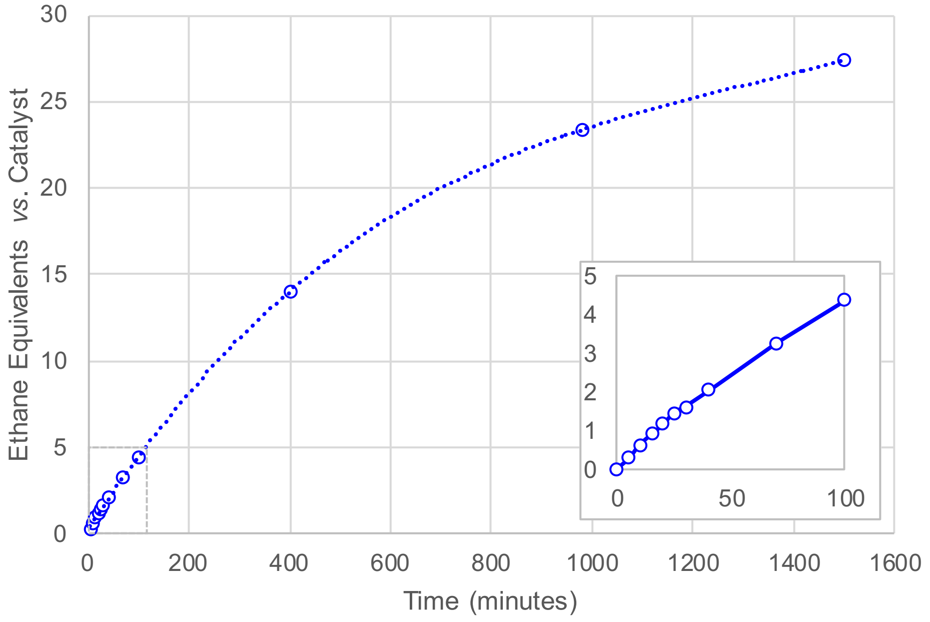

Figure S37: Ethane equivalents generated *vs.* time, determined by ^{1}H NMR integrals (400 MHz, CD_2Cl_2, 298 K).

1.22. Electrospray Mass Spectrometry; Comparison of Complexes 3 and 3-d_n

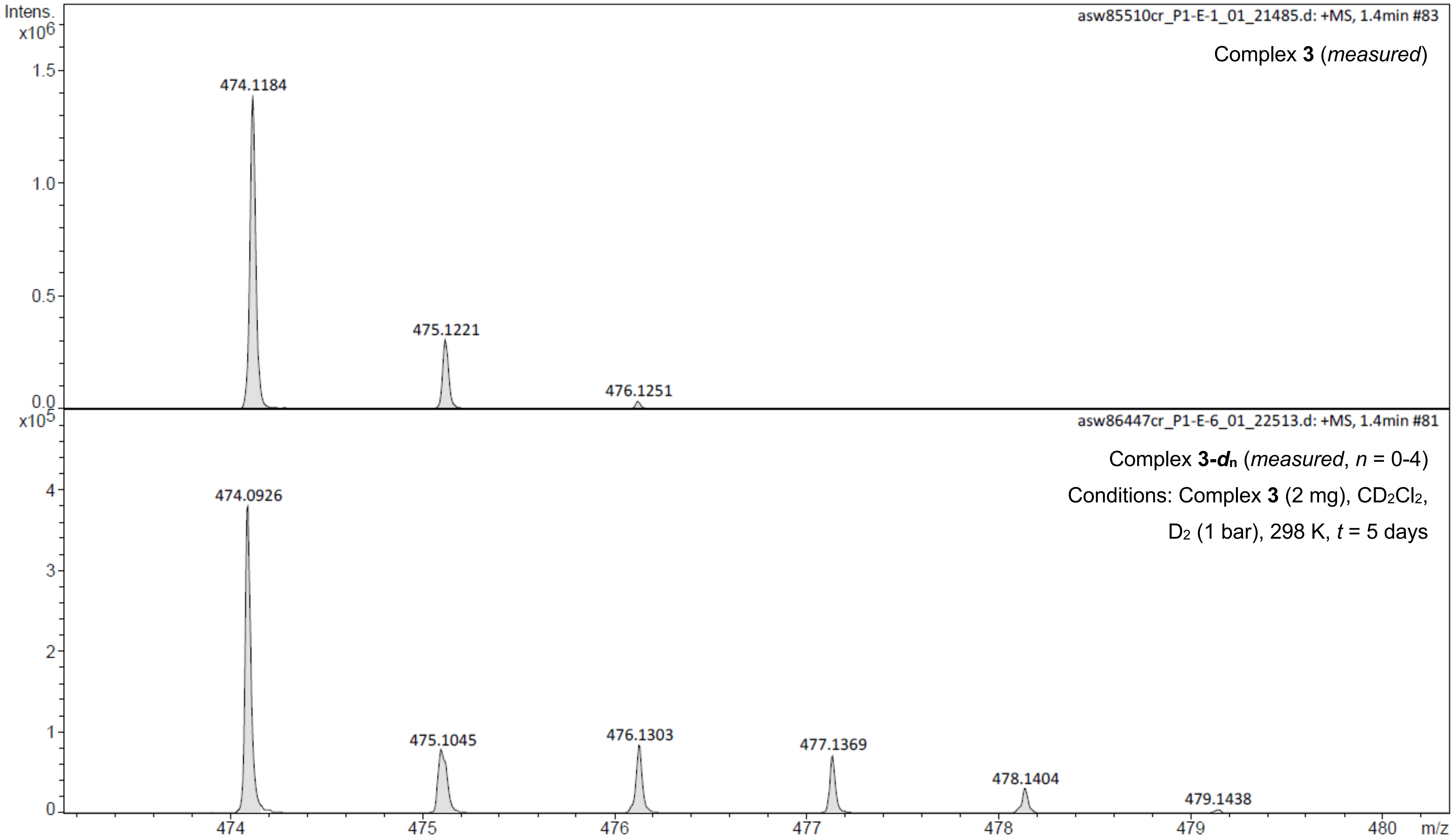

Figure S38: ESI spectra of complex **3** (top) and **3-*d*$_n$** (bottom).

2. References

[1] Solvent-free anhydrous Li+, Na+ and K+ salts of $[B(3,5\text{-}(CF_3)_2C_6H_3)_4]^-$, $[BAr^F{}_4]^-$ Improved synthesis and solid-state structures, A. J. Martínez-Martínez, A. S. Weller, *Dalton Transactions*, **2019**, *48*, 3551–3554.

[2] $[Rh_7(P^iPr_3)_6H_{18}][BAr^F{}_4]_2$: A Molecular Rh(111) Surface Decorated with 18 Hydrogen Atoms, S. K. Brayshaw, J. C. Green, R. Edge, E. J. L. McInnes, P. R. Raithby, J. E. Warren, A. S. Weller, *Angewandte Chemie International Edition*, **2007**, *46*, 7844–7848.

[3] Formation of Stable trans-Dihydride Ruthenium(II) and 16-Electron Ruthenium(0) Complexes Based on Phosphinite PONOP Pincer Ligands. Reactivity toward Water and Electrophiles, H. Salem, L. J. W. Shimon, Y. Diskin-Posner, G. Leitus, Y. Ben-David, D. Milstein, *Organometallics*, **2009**, *28*, 4791–4806.
